# Inferring TF activation order in time series scRNA-Seq studies

**DOI:** 10.1371/journal.pcbi.1007644

**Published:** 2020-02-18

**Authors:** Chieh Lin, Jun Ding, Ziv Bar-Joseph

**Affiliations:** 1 Machine Learning Department, School of Computer Science, Carnegie Mellon University, Pittsburgh, Pennsylvania, United States of America; 2 Computational Biology Department, School of Computer Science, Carnegie Mellon University, Pittsburgh, Pennsylvania, United States of America; Katholieke Universiteit Leuven Centrum Menselijke Erfelijkheid, BELGIUM

## Abstract

Methods for the analysis of time series single cell expression data (scRNA-Seq) either do not utilize information about transcription factors (TFs) and their targets or only study these as a post-processing step. Using such information can both, improve the accuracy of the reconstructed model and cell assignments, while at the same time provide information on how and when the process is regulated. We developed the Continuous-State Hidden Markov Models TF (CSHMM-TF) method which integrates probabilistic modeling of scRNA-Seq data with the ability to assign TFs to specific activation points in the model. TFs are assumed to influence the emission probabilities for cells assigned to later time points allowing us to identify not just the TFs controlling each path but also their order of activation. We tested CSHMM-TF on several mouse and human datasets. As we show, the method was able to identify known and novel TFs for all processes, assigned time of activation agrees with both expression information and prior knowledge and combinatorial predictions are supported by known interactions. We also show that CSHMM-TF improves upon prior methods that do not utilize TF-gene interaction.

## Author summary

An important attribute of time series single cell RNA-Seq (scRNA-Seq) data, is the ability to infer continuous trajectories of genes based on orderings of the cells. While several methods have been developed for ordering cells and inferring such trajectories, to date it was not possible to use these to infer the temporal activity of several key TFs. These TFs are are only post-transcriptionally regulated and so their expression does not provide complete information on their activity. To address this we developed the Continuous-State Hidden Markov Models TF (CSHMM-TF) methods that assigns continuous activation time to TFs based on both, their expression and the expression of their targets. Applying our method to several time series scRNA-Seq datasets we show that it correctly identifies the key regulators for the processes being studied. We analyze the temporal assignments for these TFs and show that they provide new insights about combinatorial regulation and the ordering of TF activation. We used several complementary sources to validate some of these predictions and discuss a number of other novel suggestions based on the method. As we show, the method is able to scale to large and noisy datasets and so is appropriate for several studies utilizing time series scRNA-Seq data.

## Introduction

Single cell RNA-Seq data (scRNA-Seq) has been used over the last few years to study several developmental and temporal processes [1–3]. These include cell differentiation studies [3, 4], in-vivo studies of developing animals [5] and response studies [2]. In all cases cells are usually sampled at specific intervals, RNA is extracted and sequenced, and expression profiles are determined. Using these expression profiles researchers then aim to reconstruct branching and cell fate decision models that underlie developmental processes.

In scRNA-Seq cells that are profiled cannot be further traced. Thus, to infer the trajectories which underlie these processes researchers often rely on computational methods that link expression profiles from different cells. Several methods have been developed for such analysis including methods that are based on dimensionality reduction followed by the construction of trees or graphs in the resulting reduced dimension space (for example, DPT [6], scTDA [7], PCA analysis [3], Monocle 2 [1], Wanderlust [8]), GPLVM [9, 10], Slingshot [11], and PAGA [12] and probabilistic methods that utilize the entire expression space such as SCUBA [13] and TASIC [14]). More recent work has also attempted to associate transcription factors (TF) with specific branching events to determine regulators of the reconstructed paths [15–17].

While the above methods successfully identify paths and branching events, and some can identify key TFs, the integration of TFs with the scRNA-Seq data has not reached its full potential. Several methods have been developed to integrate *bulk* time series gene expression data with protein-DNA interaction data, but these can only place TFs at a discrete, and often small, number of time points making it hard to determine the precise activation order and the combinatorial interactions involved [18]. A number of methods were recently proposed for identifying TF-gene interactions using scRNA-Seq data which can allow for more continuous assignments. However, most of these methods perform such assignments as a post-processing step [16, 17, 19] making it hard to utilize the information for improving model reconstruction and assignments. A few methods can actually integrate TFs as part of the model construction algorithm and these were indeed shown to improve upon methods that do not use this data [15]. However, these methods use a discrete state model in which TFs can only be assigned to a specific (pre-defined) time. This makes it hard to identify the exact activation time of these TFs, to infer combinatorial activity of TFs and the dynamics of TF complexes assembly.

**Fig 1:**
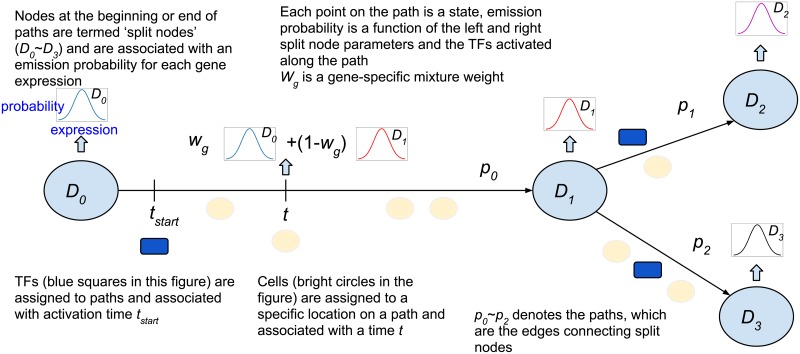
CSHMM-TF model structure and parameters. The figure presents the assignments of cells and TFs to the reconstructed branching model for the process studies. Each edge (path) represents a set of infinite states parameterized by the path number and the location along the path. We use a function based on parameters learned for the split nodes (nodes at the start and end of each path) and TF assignments to define an emission probability. Emission probability for a gene along a path is a function of the location of the state and prior TFs (*t* and *t*_*start*_) and a gene specific parameter *k* which controls the rate of change of its expression along the path. Split nodes are locations where paths split and are associated with a branch (transition) probability. The t_start parameter defines the TF activation time for a specific TF associated with the path. Cell assignment to paths is determined by the emission probabilities and the expression of specific TF targets for the TFs associated with the path. *w* is a vector of *gene-specific* mixture weight, where the weights are a non linear function which depends on (*t* and *t*_*start*_). See text for more details.

To address these issues we extended a previous method we developed for modeling dynamic scRNA-Seq branching data which was based on Continuous State Hidden Markov Models (CSHMMs) [20]. Similar to regular HMMs, CSHMMs are defined by states and transition probabilities. However, unlike traditional HMMs they have infinitely many states and so can be used to represent continuous time. The continuous states are used to determine assignment of cells to paths in the model (Fig 1) and transition probabilities are used to denote branching of cells to different fates [21]. Here we extend this model to take into account TF-gene interaction as well. We formulate an new CSHMM model (termed CSHMM-TF) in which the regulation by TFs influences the emission probabilities of the different paths. Using the revised model we associate TFs with different model paths and identify a specific activation time along the path for the different TFs. Applying our CSHMM-TF to several mouse and human scRNA-Seq datasets, we show that by using this information the resulting models are more accurate compared to models that do not use TF-gene interaction information. We also discuss the combinatorial aspects of TF regulation and show that many of the TFs assigned to the same paths are indeed working together to regulate genes. Finally, we study the dynamic of TFs activation by looking at early and late TFs for the same path (or genes) and use this to raise novel hypotheses regarding TF activation order.

## Results

To infer dynamic continuous models for both cell ordering and TF activation we developed the Continuous State Hidden Markov Models Transcription Factor (CSHMM-TF) method. An overview of the model is presented in Fig 1. We start by learning an initial branching structure (which can be modified as part of the iterative algorithm). Each edge (path) represents a set of infinite states parameterized by the path number and the location along the path. Cells are assigned to these states leading to a continuous ordering of cells along the paths. Paths can diverge at split nodes (representing a split leading to two or more different cell types) and a transition probability is inferred based on the fraction of cells assigned to each of the diverging branches. Emission probability for a gene along a path is a function of the location of the state on the path (which accounts for global gene expression in that cell) and the timing of the set of TFs assigned to the path. Specifically, we infer an activation time for some of the TFs and assign a TF specific function for their activity. We use the TF specific time and function to determine the expected expression of its targets in each point along the path. When computing emission probabilities for cells we place a larger weight on the *targets* of these TFs leading to selection of cells that are more likely regulated by them. This process iterates between model revisions, cell assignments and TF assignment until convergence. When the model converges we obtain a specific location for each cell on one of the paths and a time of activation for TFs identified.

### Application of CSHMM-TF to time series scRNA-Seq data

We applied CSHMM-TF to several time series scRNA-Seq datasets. The first is a human liver dataset with 765 cells, 19K genes, collected at 4 developmental stages [22]. The second studies human skeletal muscle myoblasts and contains 271 cells, 13K genes and 4 time points [1]. The third is from mouse and looks at differentiation of medial ganglionic eminences (MGC) to the Cortex [23]. This dataset contains ∼ 21K cells, ∼10K genes and 3 time points. The fourth is mouse embryonic fibroblasts (MEF) reprogramming to neurons [4]. It contains 252 cells, 12K genes and 4 time points. The fifth is a lung development dataset with 152 cells, 15K genes and 3 time points [3]. See S1 Appendix Supporting methods for more details about each of these datasets.

**Fig 2:**
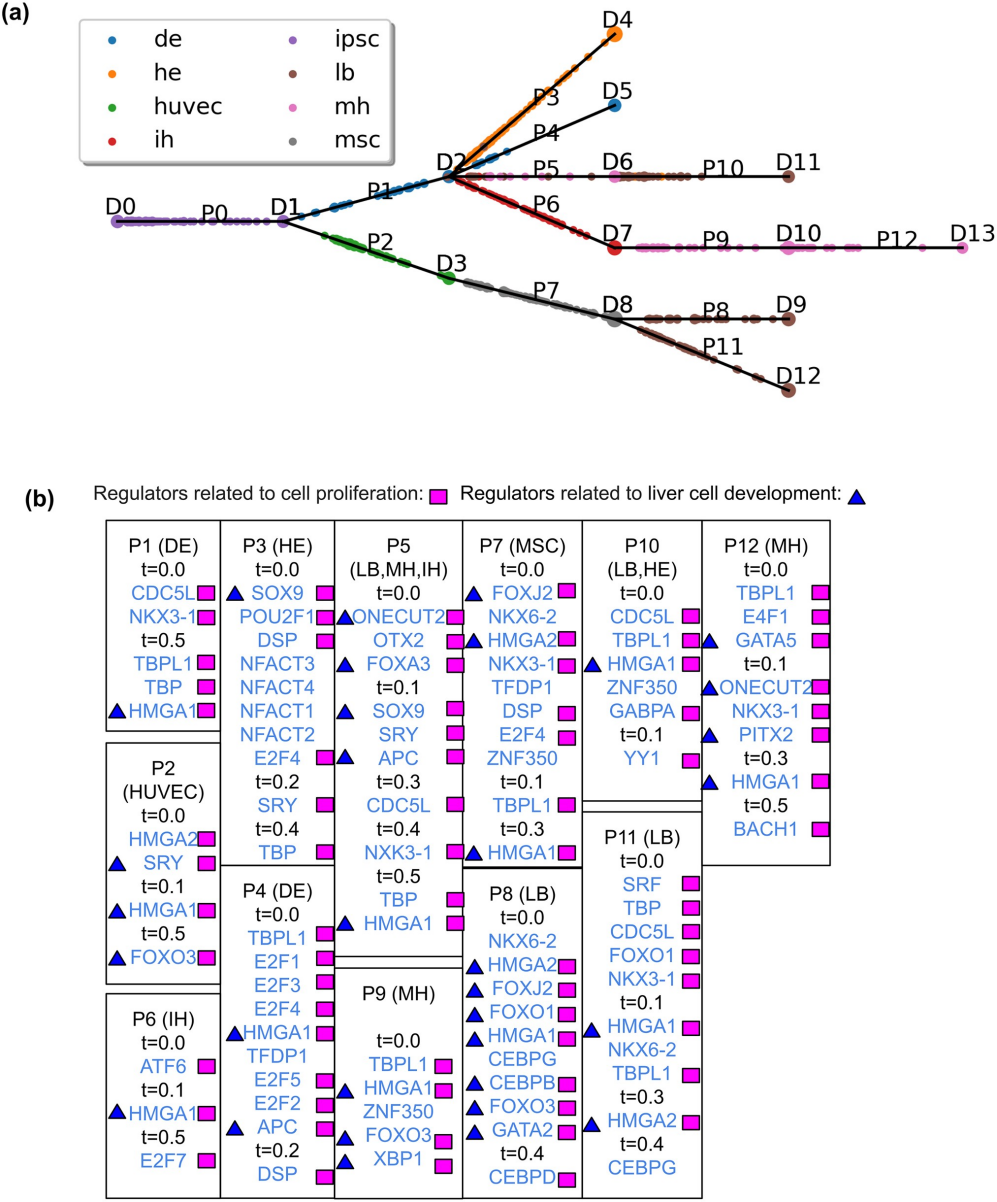
CSHMM-TF result for the liver dataset. (a) CSHMM-TF structure and continuous cell assignment for the liver dataset. D nodes are split nodes and p edges are paths as shown in Fig 1. Each circle on a path represents cells assigned to a state on that path. The bigger the circle the more cells are assigned to this state. Cells are colored based on the cell type / time point assigned to them in the original paper. (b) TF assignments by CSHMM-TF for the liver dataset. We highlight known functional roles for several TFs. Path names (DE, LB etc.) are based on annotated cells assigned to that path in the figure above. Full names of cell types can be found on S1 Appendix Supporting methods of data collection and processing.

**Fig 3:**
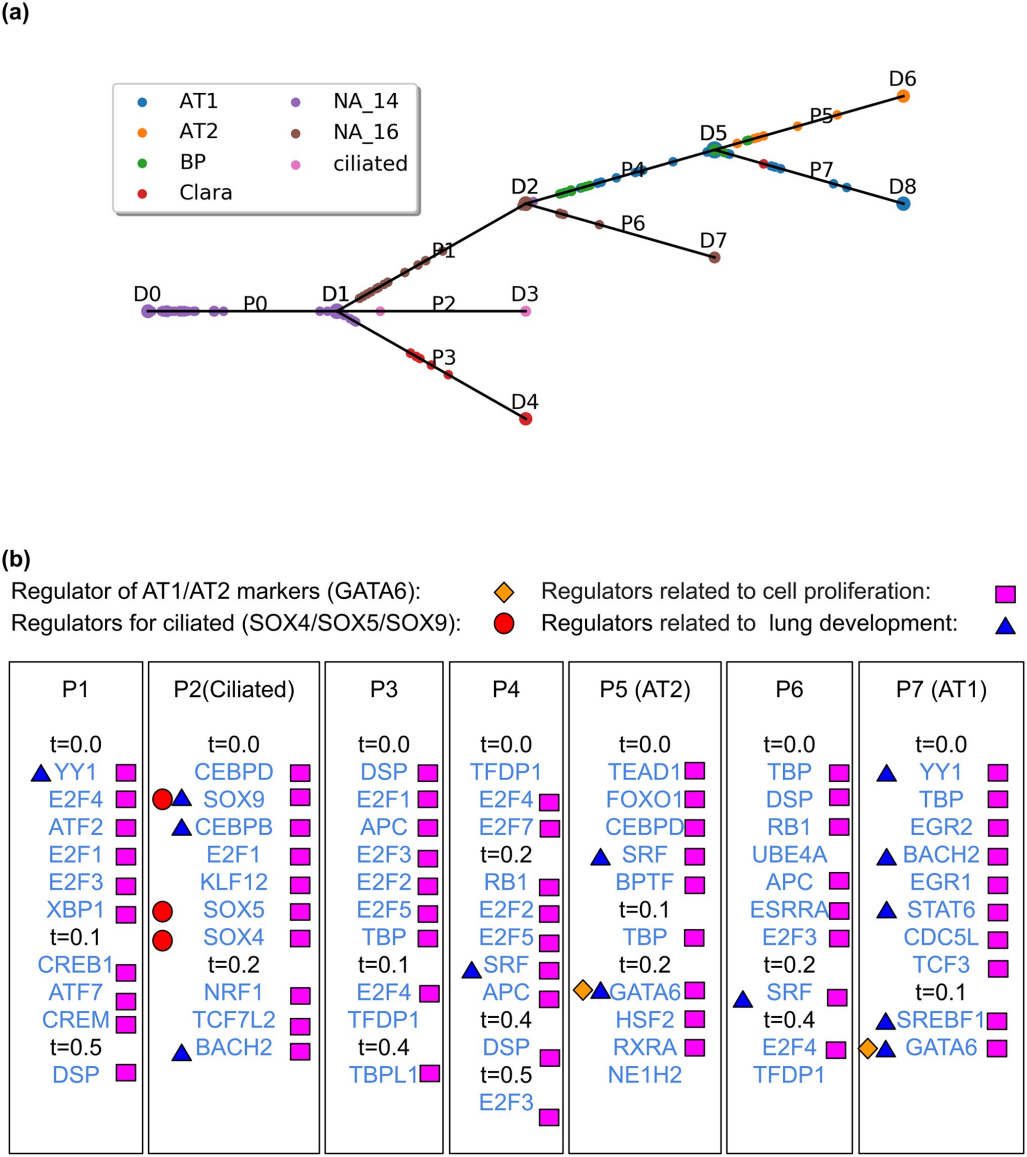
CSHMM-TF result for the lung development dataset. (a) CSHMM-TF structure and continuous cell assignment for lung development dataset. Notations are similar to the ones described in Fig 2 (b) TF assignments to each path by CSHMM-TF. We highlight known functional roles for several TFs. Path names (Ciliated, AT1 etc.) are based on annotated cells assigned to that path in the figure above.

Figs 2 and 3 present the resulting CSHMM-TF models for the human liver data and the mouse lung developmental data with TF assignments. As can be seen in these figures, unlike prior methods that assign TFs to discrete branch points only [15, 24–26], CSHMM-TF can infer a more refined time for the activation of TFs. This helps improve the assignment of cells to different paths, to infer combinatorial TF regulation and to determine TF ordering as we show below. See also Figure A-C, E and Table C, E in S1 Appendix for results for the MEF reprogramming, myoblasts differentiation, and the cortex differentiation datasets, respectfully.

The reconstructed trajectories for the liver dataset (Fig 2(a)), correctly reconstruct the relationship of induced Pluripotent Stem Cells (iPSC) → DE (definitive endoderm) → HE (hepatic endoderm) and IH (immature hepatoblast-like) → MH (mature hepatocyte-like). For the lung dataset (Fig 3(a)), CSHMM-TF correctly assigns cells, based on their known types, to terminal paths (ciliated, Clara, AT1 and AT2). Progenitor cells and BP cells are also correctly assigned to earlier paths.

### Assigned TFs correctly match cell types in each path

Figs 2 and 3(b) present TF assignment for CSHMM-TF for the liver and lung dataset. In the figures we highlight known functions related to development and the specific processes for several TFs. As can be seen, CSHMM-TF identifies known key regulators (Fig 2(b)). For example, FOX family TFs are identified in several paths and are known to control the formation and function of the liver [27]. HMGA1 (identified in all path except P3) and HMGA2 (identified in P7, P8, P11) are known to be involved in several developmental processes [28, 29]. ONECUT2 regulates liver development and is required for liver bud expansion [30]. CEBPB, identified for path P8 which is the path for liver bud, is the marker of early liver development and expressed in the early liver bud [31]. GATA2 is important in hepatic cell fate decision [32]. SOX9 is also related to hepatogenic differentiation [33]. SRF is essential for hypatocyte proliferation and liver function [34]. PITX2 is related to the differentiation of induced hepatic stem cells [35]. See S1 Appendix Supporting Results for a full list.

For the lung dataset, several of the TFs assigned by the model to the lung dataset are known to play important roles in lung development. These include SOX9 [36, 37], which plays an important role in tracheal and lung epithelium development, GATA6 [38, 39], a regulator for AT1/AT2 cell type, SREBF1 which regulates the biological process of perinatal lung maturation [40], STAT6 which can serve as a therapeutic target for preventing pulmonary hypoplasia [41], YY1 [42], which is required in lung morphogenesis and CEBPB plays pivotal role in determining airway epithelial differentiation [43]. Others include SRF, a critical protein for pulmonary myofibroblast differentiation [44] and BACH2 which is required for the functional maturation of alveolar macrophages and pulmonary homeostasis [45]. Additionally, a number of cell type specific marker genes can be identified based on their expression profiles in paths identified by CSHMM-TF. For example, AQP5 is a known marker for type 1 cells (AT1, path P7) and SFTPC, SFTPA and NKX2-1 are known markers for type 2 cells (AT2, path P5). GATA6 is the regulator for these markers [39], and is assigned to both paths by CSHMM-TF. SOX4 and SOX9 control formation of primary cilia [46] and SOX5 activates the expression of ciliary genes. All 3 TFs are correctly detected for path (ciliated path).

For both the lung and liver datasets, CSHMM-TF has also identified several TFs related to cell proliferation, as expected for developing tissues and organs. Examples are shown in the figures and the S1 Appendix Supporting results. Similar results for the neuron reprogramming dataset are also available in the S1 Appendix Supporting results.

### Verifying predicted TF activation time

**Fig 4:**
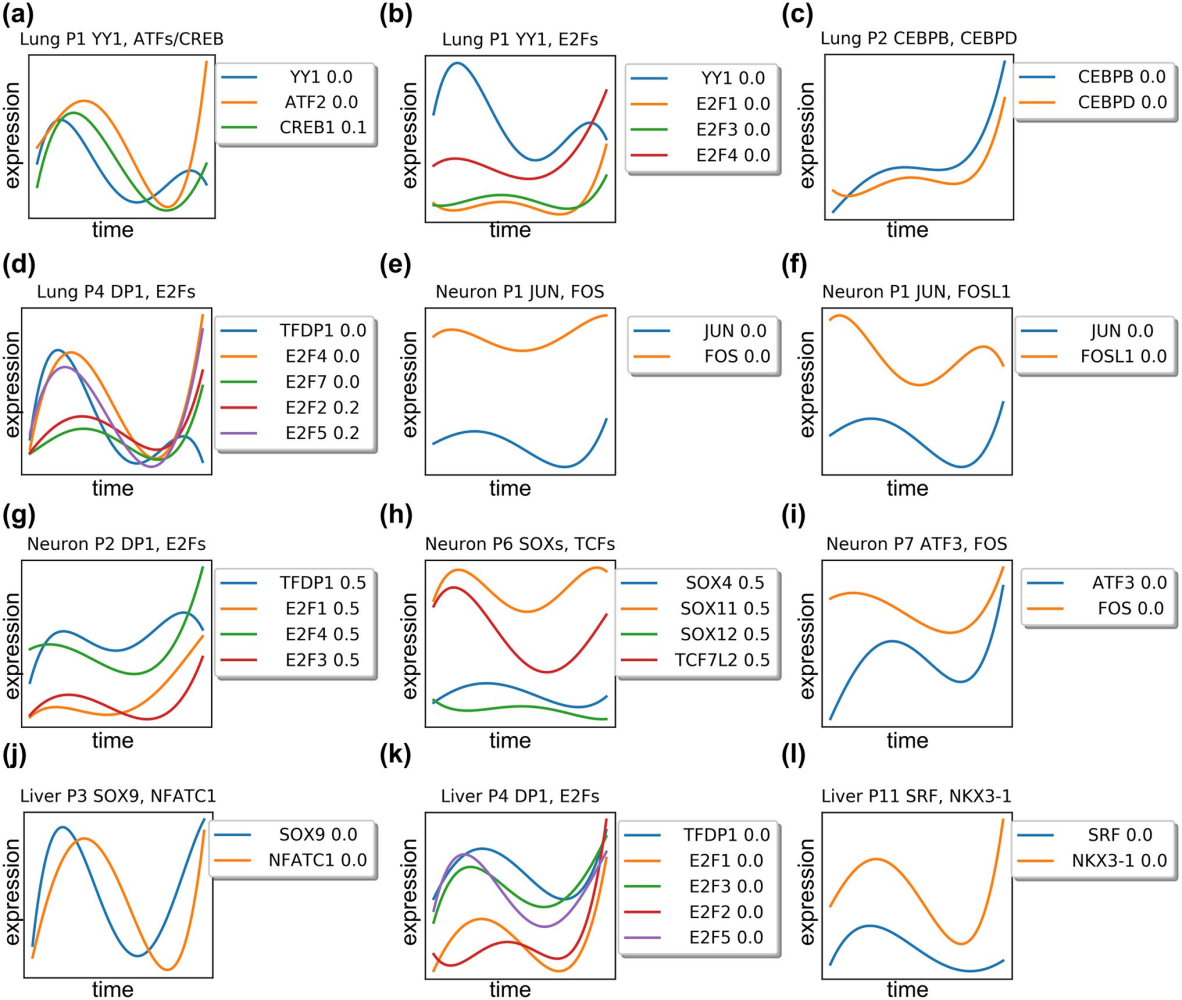
Expression profiles for top TFs assigned by the method to the lung, neuron, and liver reconstructed models. Each figure plots the expression TFs predicted to co-regulate a specific path. Each figure legend denotes the color and the *time* assignment for each TF. Profiles for TFs are the MLE estimates for these TFs expression values based on learned model parameters. (a-d) co-regulating TF expressions in lung paths. (e-i) co-regulating TF expressions in neuron paths. (j-l) co-regulating TF expressions in liver paths. See text for details.

While we observe the expression values for all genes and TFs, when learning the CSHMM-TF model we do not use the expression of the TFs. Instead, following past work [18] we determine TF activity and timing based on TF targets. This allows us to identify TFs that are post-transcriptionally regulated which are missed when only using expression data to infer activity. However, some TFs are transcriptionally regulated and we can thus use their expression profiles to validate model assignments. Specifically, since TF expression levels and protein-protein interactions are not used to infer their targets, we use them for model validation. Fig 4 presents expression profiles smoothed by 4-degree polynomial for top assigned TFs based on p-values from binomial test in the lung, neuron, and liver models. Each figure legend denotes the color and the time assignment for TFs.

Several of these profiles agree with both their time assignment and their relationship to other TFs assigned to the same paths. For example, the transcriptional repressor protein YY1 is known to directly interact with members of the ATF/CREB family of transcription factors [47]. These TFs are all assigned to path P1 with YY1 being up-regulated earlier than ATF/CREB supporting model assignments (Fig 4(a)). Similarly, interactions between YY1 and E2F genes was previously noted [48, 49] and indeed both are assigned to path P1 (Fig 4(b)). CEBPB/CEBPD, known to form a heterodimers [50] are both correctly assigned to the same time (Fig 4(c)). Similarly, E2Fs which are known to bind DP1 [51] are assigned to the same time and path (Fig 4(d), 4(g) and 4(k)). FOS and JUN can form heterodimers [52] and are also assigned the same activation time (Fig 4(e) and 4(f)). SOX genes are known to modulate beta-catenin/TCF activity [53]. Our model assigning all of them to the same time in path P6 of the neuron data, though expression analysis shows that sox11 is slightly ahead of TCF7 (Fig 4(h)). ATF3 is a known co-factor of c-Fos and both are correctly assigned to the same time (Fig 4(i)). In addition, SOX9 is known to be the downstream target of NFATC1 [54], and CSHMM-TF identified both of them in the same path and assign them at the same time point (Fig 4(j)). Finally, SRF are known to form a physical complex with NKX3-1 [55], and both of them are assigned at the same path with same time (Fig 4(l)). In Table F in S1 Appendix, we present the Spearman correlations for the expression of predicted TF pairs. As can be seen, overall the high correlations support the assignments of CSHMM-TF.

### TF interactions further support TF assignment times

In addition to the support provided by the analysis of expression profiles we looked at known interactions between TFs to determine whether TFs assigned by CSHMM-TF to the same path (either at the same or different times) are indeed known to interact. For this, we determined the number of protein-protein interactions (PPI) or regulatory interactions in each paths and compared these to random TF sets of the same size. We have further divided the analysis to determine the significance of interactions within and between a specific time assignment (early-early, late-late, or early-late where early is defined as an assignment to the branching point (0) and late as everything after that).

We searched for interactions for all 5 models in the TcoF-DB database [56], which contains transcription factor interactions for human and mouse. Results are presented in Table 1. Each dataset is represented by 3 rows: The first displays the number of interactions in the TcoF-DB in all paths, divided by the number of all combinations in all paths. Take the lung data as an example, there are 257 TFs in the dataset, so there could be 257*256/2 = 32896 possible TF interactions, but only 960 of these interactions are found in the TcoF-DB database. For the #A vs A column, the numerator is the sum of the number of interactions found in TcoF-DB, while the denominator is the sum of all possible interactions in each path (in this dataset we have identified top 10 TFs in each path, so this number becomes 10*9/2 * (7 paths) = 315). For the second row of each dataset, we just calculated the ratio based on the numbers in the first row. For the third row, we calculated the p-value based on hypergeometric test compared to the #total column.

**Table 1:** Analysis of predicted TF-TF interactions based on the TcoF database. Abbreviations: total: all possible interactions in a dataset, A: all TFs assigned to each path, E: early TFs in each of the paths, L: late TFs. For each dataset we present 3 rows: number of combinations, ratio and p-value.

Dataset	#of TF	#total	#A vs A	#E vs E	#L vs L	#E vs L
Liver #comb	252	1021/31626	20/342	11/166	2/48	7 / 128
Liver ratio		0.032	0.058	0.066	0.042	0.055
Liver-p-value		X	3.99E-03	7.85E-03	2.02E-01	5.60E-02
Lung #comb	257	960/32896	30/315	8/119	5/47	17/149
Lung ratio		0.029	0.095	0.067	0.106	0.114
Lung p-value		X	4.56E-09	8.24E-03	2.35E-03	3.91E-07
Cortical #comb	157	423/12246	19/291	9/144	0/33	10 / 114
Cortical ratio		0.035	0.065	0.063	0.000	0.088
Cortical-p-value		X	2.72E-03	2.76E-02	X	1.93E-03
Neuron #comb	208	873/21528	30/351	16/90	8/85	6/176
Neuron ratio		0.040	0.085	0.17	0.094	0.034
Neuron p-value		X	4.47E-05	1.07E-07	7.47E-03	X
Myoblast #comb	230	875/26335	49/447	45/408	0/3	4/36
Myoblast ratio		0.033	0.109	0.111	0.000	0.111
Myoblast-p-value		X	7.18E-14	5.50E-13	X	6.42E-03

Overall, we see very significant enrichment for interactions between TFs assigned to the same path. For most datasets we also see significant enrichment for interaction for ‘early TFs’. These are TFs that are assigned to the initial part of the path (usually those that regulate a large number of genes in the path) and as shown above in many cases represent proteins that are involved in complexes that jointly regulate a large number of genes. However, interestingly we also find for some of the datasets (most notably the mouse lung data) a strong enrichment for early-late interactions. These interactions likely represent a late TF activation or recruitment by an earlier TF. The fact that many of them are known interactions indicate that our model, using scRNA-Seq data, is indeed able to identify the specific timing of the regulation of the different TFs which are usually all assigned to the same time.

### Comparison to other methods

We compared CSHMM-TF with several prior methods for trajectory inference that do not utilize TF-gene interaction data. For this we looked at the accuracy of the reconstructed trajectories and cell assignments as well as on the inference of TFs and their order.

Figure B in S1 Appendix presents a comparisons for the lung and neuron datasets between CSHMM-TF and several prior methods for pseudo-time inference including PCA [3], TSNE, GPLVM following PCA [57], Monocle 2 [1, 58], Slingshot [11], and PAGA [12]. Note that, although PCA and TSNE are not cell trajectory reconstruction methods, a number of previous time series scRNA-Seq analysis papers have used these methods to discuss trajectories [3, 16]. In addition, several of the trajectory assignment methods only work on the reduced dimension representation (including GPLVM and slingshot) and so we plot the results for these methods as well.

As the figure shows, for a number of cell types these methods were unable to fully reconstruct known developmental trajectories.

For example, while PCA and TSNE, were able to identify clusters for some cell types in both the lung and neuron data, they were unable to reconstruct the correct trajectories and also mix a number of different cell types correctly assigned by CSHMM-TF. GPLVM correctly orders cells along a pseudotime, however, it is unable to determine branching models. Monocle 2 is able to reconstruct cell trajectories, however it only found a single split for these datasets and also mixed cell types that CSHMM-TF correctly separated into unique branches. Slingshot is able to order cells along a pseudotime but it did not identify any branch point for the lung data. For the neural data it correctly separates the MEF and neuron cells, but is unable to infer a correct trajectory along the different cell types (in fact, one of its trajectories ends with d2_induced which is an intermediate cell type). As for PAGA, while it correctly clusters cell types, it does not seem to provide any clear trajectory for the cells or clusters. For both datasets PAGA produces a set of weakly connected cliques making it hard to infer the branching.

To compare the results of CSHMM-TF with CSHMM that does not utilize TF-gene interactions, we developed a quantitative measure which calculates the accuracy of the ordering inferred by the two methods (S1 Appendix Supporting Methods). We used this to compare the two methods on three of the datasets analyzed in this paper: lung, neuron and liver. Results are shown in Table H in S1 Appendix. As can be seen, CSHMM-TF assignments are in better agreement with known cell differentiation stages when compared to CSHMM for all three datasets. In some of them the improvement is small (1-2%) while for the lung dataset, the improvement is about 9%. To further study the usefulness of the TF-gene interaction information we have also compared CSHMM-TF to a version that uses random TF-gene assignments. Again, we see a decrease in performance when not using the correct TF-gene interactions (Table H in S1 Appendix). For the random assignments we also determined the number of significant TFs identified by CSHMM-TF. As can be seen in Table I in S1 Appendix, random TF-gene interactions lead to much fewer significant TFs indicating that, as we assumed in the model, several co-regulated genes are assigned to the same paths by CSHMM-TF.

As mentioned above, most prior methods do not attempt to model regulation by TFs. However, a few do, and so we next compared CSHMM-TF to two prior methods for TF assignments using the liver dataset. The first is SCDIFF [15], which, unlike our method does not provide continuous assignment for cells. The second is based on post-processing assignment of TFs following model reconstruction [16, 17, 19]. These methods perform t-test for the expressions of TFs between each path and its parent path and use a p-value cutoff to select differentially expressed (DE) TFs. Here, we use the DE method as a post processing step following CSHMM analysis for comparison. Table A-B in S1 Appendix present the resulting TFs selected by SCDIFF and the DE method. For both methods we select the top 10 TFs for each path and compare these to the top 10 CSHMM-TF predictions. While we see some overlap (HMGA1, HMGA2 and PITX2) between TFs identified by the DE method, and those identified by CSHMM-TF, all other liver TFs identified by CSHMM-TF which were discussed are missed by the DE method. Similarly, we see a number of known liver development TFs that were identified by CSHMM-TF but missed by SCDIFF including ONECUT2 at P5 [30], APC [59] at P4, and SOX9 [33] at P3 and P5.

### Scalability and robustness of CSHMM-TF

While some recent scRNA-Seq studies profile thousands of cells, very few large time series scRNA-Seq datasets are currently available. One of the datasets we analyzed, which studied mouse cortical development is quite large (∼21K cells, ∼10K genes) [23]. As we have shown in Figure E and Table E in S1 Appendix, CSHMM-TF can be successfully applied to such data. Total runtime for this dataset on a desktop with 4 cores was less than 3 days and since assignments of cells to paths are easy to parallelize, run time can be significantly reduced on a larger cluster. To test performance on slightly smaller, though better annotated, dataset we performed simulation analysis based on the liver scRNA-Seq data [22] using ∼10K cells. For this, we generated a new dataset with ∼10K cells based on the human liver data. We created 13 random cells from each original cell by randomly adding 20% dropouts (setting the expression of 20% random genes in each cell to zero). Results are presented in Figure D and Table D in S1 Appendix. Run time on a desktop is about 9 hours for one EM iteration with the total run time of less than 2 days. We have also compared the accuracy of the resulting model to the original model (based on a smaller data size) and found them to be comparable. See S1 Appendix Supporting Results for complete details.

## Discussion

While several methods have been developed to reconstruct developmental models based on time series scRNA-Seq data, very few of these utilize information about TF-gene interactions. Such complementary information can aid in correctly reconstructing models for development and differentiation and can help explain the regulation of the process being studied.

Here we presented CSHMM-TF a continuous-state HMM model which combines cell assignments to a developmental model with TF assignments as regulators of the process. The method utilizes a probabilistic model which helps account for noise and missing values common to scRNA-Seq data. To learn the model the method iterates between cell assignments to branches and TF assignments to specific time points. Cells assigned to paths to which TFs are assigned are assumed to have that TF active. Based on the analysis of the targets of these cells we can both, identify the regulators and improve the assignments of cells to paths.

We applied the method to several scRNA-Seq datasets from both human and mouse. As we show, the method was able to reconstruct biologically sound models for all datasets, in most cases correctly grouping cells based on known types. In contrast, several other pseudo-time scRNA-Seq analysis methods were unable to correctly reconstruct models for at least some of these studies highlighting the advantage of integrating expression and regulation data.

Beyond the construction of the models and cell assignments to specific positions, CSHMM-TF identifies several TFs as regulating key aspects of the processes. Analysis of the TFs identified for the different biological systems studied supports these assignments since many of them are known to play important roles in those process while others represent novel predictions about the regulation of specific branching events. In addition to the list of TFs, CSHMM-TF provides information about potential combinatorial and causal relationships between TFs assigned to the same path. As we showed, TFs assigned to the beginning of paths are often interacting and in some cases early and late TFs are interacting as well. In these cases CSHMM-TF provides information on the dynamics of the assembly process of TF complexes which, without the detailed trajectories provided by scRNA-Seq would have been hard to do.

CSHMM-TF can also be complimentary to current analysis methods that are based on identifying DE TFs. For the liver data, we found that PITX2, a known liver development TF [35], appears in paths P6 for the DE while it appears as regulating a later path, P12, for CSHMM-TF. This likely means that while PITX2 is first DE early, its impact and regulatory role are only observed later in the developmental process. Such joint analysis can further improve the confidence in the identified TFs.

While CSHMM-TF was successful in analyzing several biological systems, there are certainly many places where it can be improved. First, CSHMM-TF relies on a predefined list of TF-gene interactions, and this is likely incomplete preventing the method from identifying additional key TFs. In addition, while the method is able to identify interacting TFs, the model for their impact is additive and so it would be hard for this method to identify more complex relationships (for example, AND and OR types).

CSHMM-TF is implemented in python and is available from github (https://github.com/jessica1338/CSHMM-TF-for-time-series-scRNA-Seq.git). We believe that CSHMM-TF represents a useful first step in utilizing the detailed information provided by scRNA-Seq data to infer the dynamics of TF activation.

## Materials and methods

### Data collection and processing

We tested our method on five publicly available time-series scRNA-Seq datasets in human and mouse. The number of cells in the datasets ranged from 152 (mouse lung data) to ∼ 21K (mouse cortex data). Datasets were processed by removing genes with overall low expression (following [15]). Following this step the number of genes in the models ranged from 10-18K. Details about the datasets are provided in Results, and data processing information is available in the S1 Appendix Supporting methods. Details about how TF-gene interaction information is obtained is provided in S1 Appendix Supporting methods.

### CSHMM-TF formulation

Continuous State HMMs (CSHMM) differs from standard HMMs in the number of states each can have. While HMMs have a (finite) well defined set of states, CSHMM can have infinitely many states (which we use to represent continuous time of cells). CSHMM-TF extends the formulation of CSHMM for time-series scRNA-Seq data (first presented in [21]) by adding TF regulation information to each path (edge). In addition, the model also assigns the *time at which a TF is impacting its targets*. The model assigns both activators and repressor TFs. For simplicity we are using the term “TF activation” when discussing this assignment though the actual direction of the impact is calculated independently of the timing assignment and as mentioned above can be either positive or negative. Our method uses TF targets to infer TF activity since several prior studies have shown that the expression of many TFs does not adequately reflect their activation profiles as many of them are post-transcriptionally and post-transcriptionally regulated. In contrast, the activity of target genes is often a better proxy for TF activity [60]. The assignment of continuous activation time also allows the model to infer combinatorial regulatory relationships (if two TFs are assigned to regulate the same path) and in some cases to infer the order of the recruitment process for different TFs regulating the same gene. Fig 1 presents the CSHMM-TF structure. In the figure, we denote a few states as split nodes (*D*_0_ ∼ *D*_3_ nodes). These are the states in which cells are allowed to split to two or more branches and they represent important split stages for cell lineages. The edges between split nodes are denoted as paths (*p*_0_ ∼ *p*_2_) and each contains infinitely many states such that each point on a path corresponds to an state. States are parametrized by their location w.r.t the two split nodes at the end of the path they reside on. Each of the split nodes is associated with a branch probability *B*. For each state (including split nodes), we define an emission probability by determining parameters for a multivariate Gaussian distribution which, following previous work, assumes independence for gene specific expression levels conditioned on the state [61]. The main difference between CSHMM-TF and CSHMM is that the formulation of CSHMM-TF utilizes TF-gene interaction information to change the likelihood function of cell assignments to paths. The assignment of a TF to path, and its inferred activation time (*t*_*start*_) directly affects the emission probability of cells assigned to locations on the paths that follow the start time of the TF. To formulate the emission probabilities in CSHMM-TF we use *s*_*p*,*t*_ to represent a specific state where 0 ≤ *t* ≤ 1 is a pseudo time on path *p*(*D*_*a*_ → *D*_*b*_), and *a*, *b* are the indices of split nodes. Denote by $x_{j}^{i}$ the expression of gene *j* in cell *i*, the emission probability for gene *j* in cell *i* assigned to state *s*_*p*,*t*_ is modeled as a Gaussian distribution with mean $\mu_{j,s_{p,t}}$ and variance *σ*_*j*_:

$$x_{j}^{i}∼N\left(\mu_{j,s_{p,t}},\sigma_{j}^{2}\right),P\left(x_{j}^{i}|s_{p,t}^{i},\theta \right)=\frac{1}{\sqrt{2\pi \sigma_{j}^{2}}}\exp(-\frac{{\left(x_{j}^{i}-\mu_{j,s_{p,t}}\right)}^{2}}{2\sigma_{j}^{2}}).$$

Where

$$\begin{array}{cc} \mu_{j,s_{p,t}} & =g_{aj}\exp\left(-K_{p,j}t^{\prime }\right)+g_{bj}\left(1-\exp\left(-K_{p,j}t^{\prime }\right)\right)=g_{bj}+\left(g_{aj}-g_{bj}\right)\exp\left(-K_{p,j}t^{\prime }\right)\\ & =g_{bj}+\left(g_{aj}-g_{bj}\right)\exp\left(-K_{p,j}\max\left(0,t-t_{j,start}\right)\right) \end{array}$$ (1)

Here, *θ* is the set of model parameters (see Table 2). *g*_*aj*_ is the mean expression for gene *j* at split node *a*. We assume a continuous change in expression for a subset of the genes along a path (from left split node *g*_*a*_ to right split node *g*_*b*_ with a mixture weight *w*_*j*_ = exp(−*K*_*p*,*j*_ *t*′)). Note that this weight is gene specific and depends in part on the TFs predicted to regulate that gene. To allow different genes to change non-linearly at different rates across the path (some at the beginning while others at the end) we use a gene specific parameter *K*_*p*,*j*_ to denote the rate of change. For genes regulated by TFs that do not change at the start of the path we use *t*′ = max(0, *t* − *t*_*start*_). Here, *t* is the time assignment of the cell, *t*_*j*,*start*_ is the TF activation time for TF regulating gene *j*, which we discuss in more detail below. For genes not regulated by any TF assigned to this path, or those regulated by TFs that are activated at the start of the path, *t*′ = max(0, *t* − *t*_*start*_) is equal to *t*. We also attempted to include dropout probability using a mixture weight model in the emission probability, however, this did not change the performance of CSHMM-TF much and so is omitted here. These notations are enough to define the parameters required to specify a CSHMM-TF: *θ* = (*V*, *π*, *S*, *A*, *E*′). All symbol definitions are presented in Table 2. In S1 Appendix Supporting Methods we prove that our definition of CSHMM-TF leads to a valid continuous state HMM and also provide additional details of the definition of transition probabilities for CSHMM-TF.

**Table 2:** Parameters of the CSHMM-TF model: *θ*_*CSHMM*−*TF*_ = (*V*, *π*, *S*, *A*, *E*′).

symbol	definition
*V*	the observation alphabet $\subset R^{G}$ (the possible input set)
*π*	the initial probability for each state, $\pi_{s_{0,0}}=1$
*S*	the set of states (each path has infinitely many states) *s*_*p*,*t*_ denotes the hidden state of path *p*, pseudo time *t*
*B*	the branch probability defined on each pair of paths, ∑_*j*∈*P*_ *B*_*i*,*j*_ = 1, 0 ≤ *B*_*i*,*j*_ ≤ 1 ∀*i*, *j* ∈ *P*
*A*	the transition probability defined on any pair of states $s_{p_{i},t_{i}}$ and $s_{p_{j},t_{j}}$
*E*′ = (*K*, *g*, *σ*^2^, Ω, Φ)	the parameters associated with emission probability for a given state
*K*	$K=\left\{K_{1},...,K_{\left|P\right|}\right\}\subset R^{G}$, *K*_*p*,*j*_ denotes the gene changing speed for gene *j* at path *p*
*g*	$g=\left\{g_{1},...,g_{\left|D\right|}\right\}\subset R^{G}$, *g*_*d*,*j*_ denotes the mean gene expression of gene *j* for split nodes *d* *g*_*d*_ denotes the mean gene expression vector of split node *d*
$\sigma^{2}\subset R^{G}$	the variance vector for genes
$\Omega \subset R^{G\times |F|}$	the matrix where each entry Ω_*i*,*j*_ is 0 or 1 denoting whether gene *i* is regulated by TF *j* or not
$\Phi \subset R^{\left|P|\times |F\right|}$	the matrix where each entry Φ_*i*,*j*_ denoting the relationship of path *i* and TF *j*. Where -1 means no relationship, 0 ≤ *t*_*start*_ ≤ 0.5 means TF *j* is assigned to path *i* with time *t*_*start*_
*D*	the set of split points
*P*	the set of paths
*G*	the number of genes (dimension of data)
*F*	the set of TFs
λ_*g*_	the hyper parameter for the L1 regularization that controls the sparsity of Δ*g* for every path *p*

### Assigning regulating TFs to each path

To predict regulating TFs for each path we extend methods that only allow discrete time assignments to TF activity [15]. We first remove TFs that are expressed in less than 20% of cells in the path. Next, we determine differentially expressed (DE) genes by performing a t-test between cells assigned to the current and parent path (S1 Appendix Supporting Methods). After we identify the set of DE genes, we use the TF-target information (Ω parameter) obtained from [24, 62] to calculate the p-value (based on hyper-geometric distribution) for each TF for this path. Details about the how the TF-target information is provided in S1 Appendix Supporting Methods. We keep TFs with a p-value ≤ 0.05 (p-value obtained by binomial test) with an upper bound of 10 TF for each path. The method for assigning TFs in each path is presented in S1 Appendix Supporting Methods (in the section “Assigning pseudo time to TF regulating a path”).

### Adjusting regularization parameters based on TF assignments

We assume that most genes do not change in a specific path (i.e. developmental branching is only affecting a subset of the genes). Based on this we regularize the gene expression difference vector (Δ*g*) which represent the change in expression for each gene between the two nodes that define a path (start and end). We use a L1 regularization with parameter λ_*g*_, where larger λ_*g*_ means more strict regulation. To incorporate TF information to this regularization (given our assumption that genes regulated by path specific TFs are more likely to change in that path) we use instead $\frac{\lambda_{g}}{1+\alpha_{p,j}}$ as the regularization term. Here *α*_*p*,*j*_ is the probability that the expression of gene *j* will change along path *p* (and so the higher the probability the lower the regularization for gene *j*). *α*_*p*,*j*_ is estimated by fitting a logistic regression model for all genes regulated by TFs on path *p*. Such changes in the regularization parameters allow genes that are targets of assigned TFs to change more than other genes for which no explanation for change in expression is determined by the model.

### Likelihood function for the CSHMM-TF model

We use the following notations: we assume we have *N* cells. Let *X*^*i*^ denote the expression profile of cell *i* and let $y^{i}=s_{p,t}^{i}$ be the hidden state denoting that cell *i* is assigned to path *p* with pseudo time *t*. Δ*g*_*p*_ is the difference vector for the expression values at the endpoints of path *p*. Using notations defined above the log-likelihood with L1 regularization term is:

$$\begin{array}{c} l\left(\theta |X,Y\right)=\sum_{i=1}^{N}\log P\left(X^{i},y^{i}|\theta \right)+\log\left(\text{L1}\;\text{regularization}\;\text{term}\right)\\ =\sum_{i=1}^{N}\sum_{j=1}^{G}\log P\left(x_{j}^{i}|s_{p,t}^{i},\theta \right)+\sum_{i=1}^{N}\log P\left(s_{p,t}^{i}|\theta \right)+\sum_{p\in P}\sum_{j=1}^{G}-\frac{\lambda_{g}}{1+\alpha_{p,j}}\left|{\left(\Delta g_{p}\right)}_{j}\right| \end{array}$$ (2)

Where,

$$\begin{array}{c} P\left(s_{p,t}^{i}|\theta \right)=\prod_{\begin{array}{c} q\in \text{branch}\;\text{probability}\\ \text{from}\;\text{root}\;\text{to}\;p \end{array}}q\;\;(\text{the}\;\text{branch}\;\text{probability}) \end{array}$$ (3)

$$\begin{array}{c} P(x_{j}^{i}|s_{p,t}^{i},\theta )\;\;\text{(the}\;\text{emission}\;\text{probability)}\\ =\frac{1}{\sqrt{2\pi \sigma_{j}^{2}}}\exp\left(-\frac{{\left(x_{j}^{i}-g_{bj}-\left(g_{aj}-g_{bj}\right)\exp\left(-K_{p,j}\max\left(0,t-t_{j,start}\right)\right)\right)}^{2}}{2\sigma_{j}^{2}}\right) \end{array}$$ (4)

Where (*g*_*a*_, *g*_*b*_) refers to the mean gene expression of the split point at both ends of a path. Briefly, the log-likelihood shown in Eq 2 contains three terms. The first, further expanded in Eq 4, represents the emission probability of each cell. Note that in this part we use a modified cell time *t*′ as we have discussed previously. The second, expanded in Eq 3, represents the penalty we use for cells assigned on later (more specific) paths. The idea is similar to prior probabilistic methods for reconstructing branching trajectories [14]: earlier stages are often less specific (higher entropy [63], while later stages (representing specific fates) have a tighter expression profile. Thus, cells that represent specific cell types will still be assigned to their correct (late) stage based on their expression profile while noisier cells would be assigned to the earlier stages. The last term in Eq 2 is the new L1 regularization term, where the L1 parameter has been replaced as we have discussed previously.

### Model initialization, learning and continuous cell assignments

**Fig 5:**
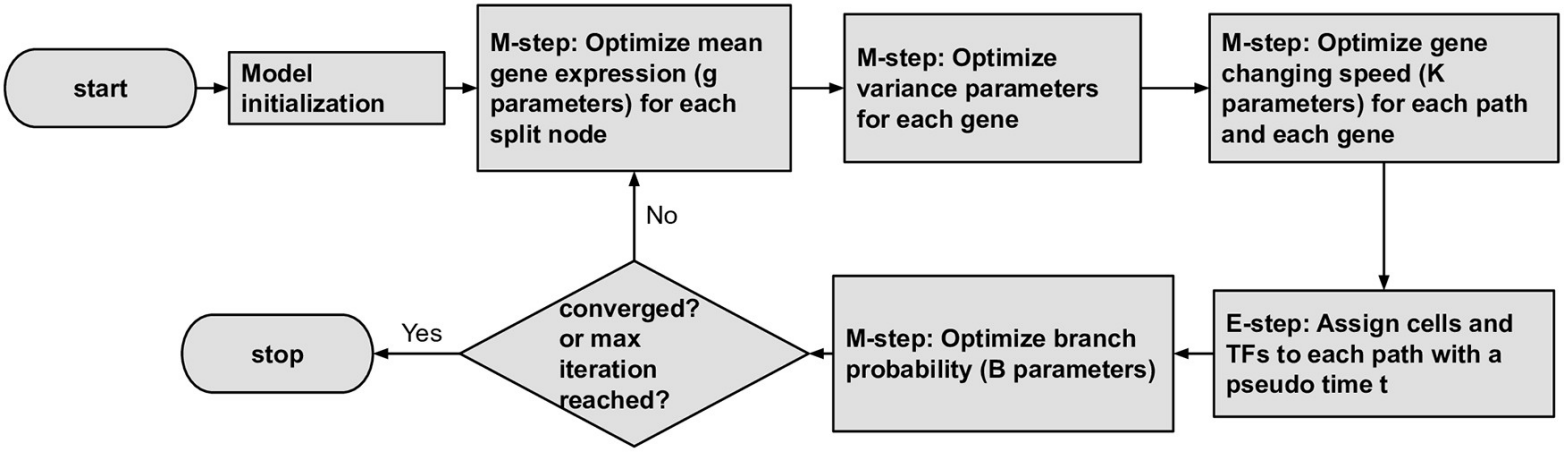
flow chart of how to iteratively learn CSHMM-TF.

For model initialization, the advantages of the SCDIFF initialization method [15] for CSHMMs have been previously discussed in [21]. Based on these results we use the same initialization for CSHMM-TF as well. Specifically, we first construct a discrete branching model based on the time-series scRNA-Seq data only. This step includes performing clustering for each time point, adjusting the level of the clusters based on time point information, and constructing a tree-branching model from the clusters. While initial assignment is based on the time information, cells can be re-assigned to different tree branches (representing other time points) as part of the iterative learning of the model. In this model, which uses prior methods for pseudotime ordering (SCDIFF [15]) cells are assigned to discrete nodes rather than continuously to paths, and no TF information is used. Next, we assign cells in each internal node to a random location along the corresponding developmental path that is incoming to that node leading to an initial continuous model. Details about model initialization for CSHMM-TF are presented in S1 Appendix Supporting Methods. For model learning and continuous cell assignments, we adopt the Expectation-Maximization algorithm (EM), where in the E-step we do the continuous cell assignments; in the M-step we try to maximize the likelihood of CSHMM-TF with Maximum Likelihood Estimation (MLE) and sampling. We iterate between E-step and M-step to improve the likelihood of the model. Fig 5 presents a flowchart for the steps used when learning CSHMM-TF. Details about parameter learning for CSHMM-TF are also presented in S1 Appendix Supporting Methods.

## Supporting information

S1 Appendix Supporting methods and results. (PDF)
