# Supplementary material for: Inferring TF activation order in time series scRNA-Seq studies
Source: PLoS Comput Biol. 2020 Feb 18;16(2):e1007644. doi: 10.1371/journal.pcbi.1007644 (PMC7048296; doi:10.1371/journal.pcbi.1007644)
Supplement: S1 Appendix — (PDF) [file pcbi.1007644.s001.pdf]

Appendix for "Inferring TF activation order in time series scRNA-Seq studies"

Supporting methods

Data collection and processing

We tested our method on five publicly available time-series scRNA-Seq datasets in human and mouse. These include human liver development study in which cells were followed from pluripotency in 2D culture and 3D liver buds [11], mouse lung development data [72] which profiles lung epithelial cell differentiation, mouse cortical development data [53], human skeletal muscle myoblast development data [71], and mouse neuron reprogramming data that studies the cell reprogramming trajectories from embryonic fibroblasts (MEFs) to neuron cells [73]. For the human data, we used the same processing method as TASIC [58], which keeps only the genes expressed in more than 25% of cells for further analysis. Mouse neuron and mouse lung datasets were processed as suggested by the original studies: We filtered genes with either FPKM < 1 in all cells or zero variance. Next, expression values were transformed to log space. For mouse cortical data we first removed genes expressed in less than 5% of cells. Then, for each cell we normalized its expression and log2-transformed all values. After these initial pre-processing, the human liver data contains 765 cells with 19K genes in 4 developmental stages (iPSC (induced pluripotent stem cells) $\rightarrow$ DE (definitive endoderm) $\rightarrow$ HE (hepatic endoderm), IH (immature hepatoblast-like) $\rightarrow$ MH (mature hepatocyte-like), LB (liver buds) and mesenchymal stem cell (MSC), Human umbilical vein endothelial cells (HUVEC)). The mouse lung dataset contains 152 cells with 15K genes measured at three time points E (Embryonic)14.5, E16.5, and E18.5. At timepoint E18.5 (though not at E14.5 and E16.5), cells were labeled with one of the following cell types: alveolar type 1 (AT1), alveolar type 2 (AT2), bipotential progenitor (BP), Clara, and Ciliated. We used the profiled time point to label cells in E14.5 and E16.5 as NA_14 and NA_16. For the mouse cortical data, we select the medial ganglionic eminences (MGE) cells and cortex cells, and this result in ~21K cells with ~10K genes and 3 time points (E (embryonic) 13.5, E18.5 and P (postnatal) 10). At E13.5 cells are labeled as MGE, which is the progenitor of cortex cells. At E18.5 and P10, all cells are labeled as cortex cells. The mouse MEF dataset is composed of 252 cells with 12K genes measured at 4 time points (0, 2, 5, 22 days). Cells were labeled with one of the following cell types: Neuron, Myocyte, and Fibroblast, MEF (mouse embryonic fibroblasts), and other progenitor-like cell types. The human skeletal muscle myoblast dataset has 271 cells with 13K genes and 4 measured time points (0, 24, 48, 72 hours). Please note that, although some of the datasets we used are well-labeled based on known markers, CSHMM-TF is purely unsupervised. Cell labels are only used for evaluation in the result section.

Details about the how the TF-target information data is obtained

Transcription factors (TF) are proteins that bind to specific DNA sequences and regulate transcription processes. Each TF activates or represses the transcription of a specific set of genes if the TF binds to the DNA location related to the genes. It has been a challenging task to identify the protein-DNA relationships for an organism. In this paper we use the information of potential targets of a set of transcription factors for human and mouse [24, 62]. This information is used to identify potential key regulators for each developmental processes. The details of how this data is obtained is described in [62]. Briefly, this data is constructed from 3 parts. In the first part, the human ChIP-Seqencing data is downloaded from ENCODE [15]. This data contain aggregated binding peaks for 148 human TFs across diverse cell lines. For each human gene, all the TFs that have transcription start sites near the gene were considered to regulate the gene. For the second part, ranked human PWM-gene predictions were obtained from [23] and each PWM was mapped to correspond TFs by using TRANSFAC [52] and JASPAR [75]. For a gene, a protein-DNA interaction was identified if the gene is in the top 100 predictions in any of the PWM for TFs. The last part is for mouse TFs, the proten-DNA interaction is derived from the second part except that a top 1000 threshold is used instead of top 100. Human gene ids were translated to mouse gene ids based on Mouse Genome Database (MGD) [5] and HUGO Gene Nomenclature Committee (HGNC) database [63].

CSHMM-TF is a valid continuous state HMM

To show that the model defined above is indeed a Continuous-State Hidden Markov Model (CSHMM) we extend the argument used by [47]. There it is shown that without including the TF information, $\theta = (V, \pi, S, A, E)$ is indeed a Continuous-State Hidden Markov Model model (CSHMM) with a properly defined initial probability (π), a transition probability function (A) that covers all states (S) and an emission probability model for each state. The above are sufficient conditions to fully define a continuous-state HMM [1]. In the new model, TF information only effects emission probability (E') and so all other correctness claims for emission and initial probability stand. As for the emission, to show that the model indeed defines a unique emission probability for each state note that the TF model introduces two additional parameters: (Ω and Φ). Ω is the matrix encoding TF-target information with $\Omega_{i,j}$ denoting if gene i is regulated by TF j. This is part of the input and so defined for all states. Φ stores the information of TF activation time with $\Phi_{i,j}$ denoting if TF j is regulating path i and the value represents the activation time. Default values for all i, j entries in this matrix are null (no impact) which means that the TF-gene info for this TF is not used by the model for this path. Only TFs for which the timing is defined are used by the model. Since these are specifically assigned in the Expectation-Maximization (EM) steps (see below) we are guaranteed that they would satisfy the requirement for a valid emission probability as required by a HMM. We thus conclude that the new model is also a valid continuous-state HMM (CSHMM).

Definition of the transition probability (A) of CSHMM-TF

CSHMM-TF adopts the same transition probability definition as CSHMM.
The transition probability $A(s_{p_1,t_1}, s_{p_2,t_2})$ for each pair of states $s_{p_1,t_1}, s_{p_2,t_2} \in S$ is defined as follows:

$$A(s_{p_1,t_1}, s_{p_2,t_2}) = 0, \text{ if } s_{p_2,t_2} \text{ is not reachable from } s_{p_1,t_1} \tag{1}$$

$$A(s_{p_1,t_1}, s_{p_2,t_2}) = 1/Z_{p_1,t_1}, \text{ if } p_2 = p_1 \text{ and } t_2 > t_1 \tag{2}$$

$$A(s_{p_1,t_1}, s_{p_2,t_2}) = \prod_{\substack{q \in \text{branch probability} \\ \text{from } p_1 \text{ to } p_2}} \frac{q}{Z_{p_1,t_1}}, \text{ if } p_2 \neq p_1, \ p_2 \text{ reachable from } p_1 \tag{3}$$

Where $s_{p,t}$ is the hidden state of cells assigned at path p with pseudo time t, Z_{p_1,t_1} is a normalizing factor for the transition probability going out of state s_{p_1,t_1} i.e..

$$Z_{p_1,t_1} = 1 - t_1 + \sum_{\substack{\text{path } p \\ \text{reachable from } p_1}} \prod_{\substack{q \in \text{branch probability} \\ \text{from } p_1 \text{ to } p}} q. \tag{4}$$

The branch probability (B) is defined on split nodes (D). The second term in equation 4 is the product of all branch probabilities of the paths from p_1 to p. For example, assume that there are two paths in between states p_1 and p: p_a and p_b. Then the second term will be $B_{p_1,p_a} * B_{p_a,p_b} * B_{p_b,p}$, where B_{p_a,p_b} refers to the branch probability for cells to transition from p_a to p_b. Note that transition probabilities integrate to 1 for each state. Also transitions and emissions only depend on the current state.

Supporting details on finding DE genes

We first using t-test between each path and its parent path to find the genes that has p-value smaller than 0.05. After that, we uses a set of log2 fold change values (0.6, 1.0, 1.5) to get three DE genes list. The reason we use a set of fold change values is because that datasets usually have different expression changes between paths therefore using a set of fold change values we will be less likely to lose the DE genes information.

Assigning pseudo time to TF regulating a path

In addition to the assignment of TFs to paths, we would also like to use the scRNA-Seq data to fine tune the specific time at which the TF exerts their influence on genes in the path. This is a major advantage of the continuous scRNA-Seq data that cannot be obtained with time series bulk data given its discrete sampling nature. To determine t_{start} for each TF / path we use a modified pseudotime $t' = t - t_{start}$ to calculate the probability of the target genes being regulated by TF, which will thus make TF have an effect on the

loss function (log-likelihood) $t_{j,start}$ is defined as the smallest activation time for the target gene j if it is regulated by multiple TFs. If no regulating TFs are detected for a gene, the $t_{j,start}$ will be defined as 0 which will have the same effect as CSHMM. The t_{start} of the TF is then set to the best value from 0 to 0.5 with the highest probability to its target genes by sampling 5 points uniformly. CSHMM-TF only allows the target gene expression starting to change after t_{start}, so setting t_{start} close to 1 will make the target gene expression not changing in the path. Therefore, we restrict $t_{start} \leq 0.5$ to make sure that the target gene expression have enough time to change. This information is then stored in parameter Φ. Note that, We have included the TF activation time t_{start} in the likelihood function so we only need to find the best value for t_{start} that makes the probability of target genes highest. The shape of the expression profile of gene j is now describe by parameter: $K_{p,j}$ (speed of changing of gene j on path p) and $t_{j,start}$ (starting time for gene j).

Model initialization

For model initialization we apply the same strategy used in SCDIFF tool [21], which construct an initial cell differentiation tree by clustering the cells at each time point, and then compute the distance of each of the clusters to the root of the tree (cells in first time point). Using this distance function clusters are assigned to different levels in the tree (where clusters in each level are significantly more distant from the root than the preceding level). Finally, each cluster (except the root cluster) at level i is connected to a parent cluster in level $i-1$ by selecting the closest cluster, in expression space, in level $i-1$. Following the initialization step of SCDIFF, we associate each cluster associated with a path (the edge connecting it to its parent). Finally, cells in each cluster are randomly assigned along the path for that cluster. Split nodes are defined for cases where two or more clusters at a specific level connect to the same cluster at the level above them. The TFs are not assigned in the model initialization step. The effect of different model initializations and how they affect the final result has been tested in [47].

Model learning and inference

We use an Expectation-Maximization (EM) algorithm to learn the parameters of the model and to infer new cell assignment. Given initial cell assignments, the branching probabilities can be easily inferred using standard Maximum Likelihood Estimation. In the following sections we discuss how to learn the emission probability parameters which, due to the K parameter requires an optimization of a non convex target function. As for cell assignment, given model parameters we assign each cell to a state $s_{p,t}$ which maximizes the log-likelihood of the resulting model. Again, since the likelihood function is not concave, determining a optimal value t for a cell assigned to path p is challenging. We will discuss a sampling strategy for solving this problem which we use to assign cells in the following sections.

Inferring cell assignments (E-step)

Given model parameters θ, we would like to assign each of the cells in our input dataset expression matrix X to a state $s_{p,t}$ which maximizes the log-likelihood. Determining a optimal value t for a cell assigned to path p is hard to be performed in closed-form because the likelihood function to t is not concave. Instead, similar to the optimization of $K_{p,j}$ parameter, we use a sampling strategy to find the best time along a path for each cell. Specifically, for each path we sample 100 points uniformly and compute the likelihood of assigning the cell to each of these points. Since the likelihood function (when model parameters are known) decomposes based on cells, this process is efficient.

Model learning (M step)

Given initial cell assignments, the branching probabilities can be easily inferred using standard Maximum Likelihood Estimation (see below).

Next, we discuss learning the emission probability parameters. For genes that change along a path, we need to learn a mean value g for split nodes and the $K_{p,j}$ parameter which encodes for each path and each gene the rate of change between the start and end expression values for that gene on that path. For K, even with a fixed mean value g for each split node, it is difficult to compute it in close form because of

non-convexity. We thus use a line search strategy to determine $K_{p,j}$. For this we compute the likelihood for 100 possible values between 0 to 10 (since $e^{-10} \approx 0$), and choose the value that achieves the maximum probability for $K_{p,j}$ (note of course that since this is a gene and path specific parameter it can be done independently for each gene / path).

As for g, let $w_j^i = \exp(-K_{p,j}t'^i)$, λ_g be the L1 sparse parameter, and Δg_p is the difference vector for the expression values at the endpoints of path p. Using notations defined above, the negative log likelihood terms that depend on g are:

$$\begin{aligned} NLL &= \sum_i^N \sum_j^G \frac{1}{2\sigma_j^2}(x_j^i - \mu_{j,s_p,t}^i)^2 + \sum_{p\in P} \sum_{j=1}^G - \frac{\lambda_g}{1+\alpha_{p,j}} |(\Delta g_p)_j| \\ &= \sum_i^N \sum_j^G \frac{1}{2\sigma_j^2}(g_{pa,j} w_j^i + g_{pb,j}(1-w_j^i) - x_j^i)^2 \\ &+ \sum_{p\in P} \sum_{j=1}^G \frac{\lambda_g}{1+\alpha_{p,j}} |(\Delta g_p)_j| \end{aligned} \tag{5}$$

where (g_{pa}, g_{pb}) refers to the mean gene expression of the split point at both ends of a path.

Since the function is convex, in CSHMM we let $\lambda_g = 1$ and use CVXPY [30, 29, 19], a disciplined convex optimization toolkit utilizing cone-spliting interior point method, to solve the linear system. Now for CSHMM-TF, we also provide another option that is glasso r package [26] to solve the L1 lasso problem because it is usually faster than CVXPY. As for the variance, since we assume that the variance σ_j of each gene j is the same across all the paths, once we have the g values we can use a standard MLE method to derive the closed-form solution for its estimation (see following supplementary section).

Details for MLE

Branch probability

First, we have the constraint that $\sum_{p_2} B_{p_1,p_2} = 1 \quad \forall p_1, p_2 \in P$. Using Lagrange multipliers we can write:

$$L(X,Y,\alpha,\theta) = \left(\sum_{i=1}^N \sum_{\substack{q \in \text{branch probability} \\ \text{from } p_1 \text{ to } p_2}} \log(q) \right) + \alpha^T (B1 - 1) \tag{6}$$

We obtain the update for B_{p_1,p_2} by setting gradient to 0

$$\frac{\partial L(X,Y,\alpha,\theta)}{\partial B_{p_1,p_2}} = 0 \Rightarrow \frac{N_{p_1,p_2}}{B_{p_1,p_2}} + \alpha_{p_1} = 0 \tag{7}$$

$$\sum_{p_2} B_{p_1,p_2} = 1 \Rightarrow \sum_{p_2} \frac{-N_{p_1,p_2}}{\alpha_{p_1}} = 1 \Rightarrow \alpha_{p_1} = \sum_{p_2} -N_{p_1,p_2} \tag{8}$$

$$\Rightarrow B_{p_1,p_2} = \frac{N_{p_1,p_2}}{\sum_{p_2} N_{p_1,p_2}} \tag{9}$$

Where N_{p_1,p_2} is the number of cells assigned to path p_2 that comes from p_1

Learning σ_j

We compute the gradient of σ_j, the variance parameter for each gene:

$$\frac{\partial}{\partial \sigma_j} \log P(X, Y|\theta) = \frac{\partial}{\partial \sigma_j} (\sum_{i=1}^{N} \sum_{j=1}^{G} \log P(x_j^i | s_{p,t}^i, \theta)) \tag{10}$$

$$= \frac{\partial}{\partial \sigma_j} (\sum_{i=1}^{N} \log N(\mu_{j,s_{p,t}^i}, \sigma_j^2)) \tag{11}$$

$$= \frac{\partial}{\partial \sigma_j} (\sum_{i=1}^{N} \log \frac{1}{\sqrt{2\pi\sigma_j^2}} \exp(-\frac{(x_j^i - \mu_{j,s_{p,t}^i})^2}{2\sigma_j^2})) \tag{12}$$

$$= \frac{\partial}{\partial \sigma_j} (\sum_{i=1}^{N} - \log(\sigma_j) - \log(\sqrt{2\pi}) - \frac{(x_j^i - \mu_{j,s_{p,t}^i})^2}{2\sigma_j^2}) \tag{13}$$

$$= \sum_{i=1}^{N} (-\frac{1}{\sigma_j} + \frac{(x_j^i - \mu_{j,s_{p,t}^i})^2}{\sigma_j^3}) \tag{14}$$

Setting gradient to 0 we have:

$$0 = \sum_{i=1}^{N} (-\frac{1}{\sigma_j} + \frac{(x_j^i - \mu_{j,s_{p,t}^i})^2}{\sigma_j^3}) \tag{15}$$

$$\Rightarrow \sigma_j^2 = \frac{\sum_{i=1}^{N} (x_j^i - \mu_{j,s_{p,t}^i})^2}{N} \tag{16}$$

Quantitative measure for comparing CSHMM and CSHMM-TF models

We developed a distance function on the cell assignments of CSHMM and CSHMM-TF models based on partial orderings ($\mathbb{P}$) defined from literature. Specifically, assumes that from literature, we know that cell type A is the parent cell type of cell type B, we denote this relationship as A→B (partial ordering). For every pair of cells (c_i, c_j) that has A→B relationship, we calculate the number of cells between c_i and c_j that are neither type A nor type B. Therefore, we assume that there are no other cell types between cell type A and B. For A→B→C relationships, we instead calculate A→B and B→C and sum them together. The total distance is the summation of each pair of cells that belongs to each pair of partial orderings. That is:

$$\text{Distance} = \sum_{(A,B)\in\mathbb{P}} \sum_{c_i\in A, c_j\in B} \sum_{c_k\notin A\cup B} \mathbb{1}_{(c_k \text{ lies between } c_i \text{ and } c_j)} \tag{17}$$

Supporting results

TFs for cell proliferation

In Result, we mentioned that most of the TFs in the lung and neuron datasets are related to cell proliferation. Examples are as follows: E2Fs [37], YY1[82], ATF2/ATF7[41], XPB1 [35], CREB/CREM [18], DSP [76], TBP/ELK1 [91], TBPL1 [84], CEBPs [40], SOX9 [78], KLFs[3], SOX5[6], SOX4 [12], NRF1[39], TCF7L2 [65], BACH2 [54], SRF[32], APC [55], RB1 [38], TEADs [48], FOXOs [4], BPTF [81], GATA6 [2], HSF2 [67], RXRs [66], ESRRA [70], EGR1/EGR2 [22], STAT6 [9], CDC5L [79], TCF3 [56], SREBFs [80], FOS/FOS-B/FOSL1/JUN/JUNB/JUND [25], GAPBs [87], EP300 [27], HSF1 [93], FOXJ2 [64], REST[88], NFIL3 [86], FLI1 [8], ETS1[60], SOX11/SOX12[46], SOX8 [85], HMGA2 [69], MAX [45], TFAP4 [17], NF1[13], ATF5 [51], ATF1 [34], NKX3-1[42], SRY[7], FOXO3[57], POU2F1[92], ONECUT2[50], OTX2[61], FOXA3[68] ATF6[33], FOXJ2[90], GATA2[74], FOXO1[77], GATA5[83], E4F1 [16], PITX2[44], BACH1[89].

TF assigned correctly for liver development dataset

Besides the TFs mentioned in Result , CSHMM-TF also identified other TFs that is related to liver development. For example, APC is related to the WNT signaling pathway in liver development [10]. XBP-1 is a transcription factor essential for hepatocyte growth [59]. GATA5 is reported to be essential in liver development in other organisms [28, 36].

TF assigned correctly for neuron reprogramming dataset

For the neuron reprogramming dataset, CSHMM-TF also identifies known key regulators for some of the cell types (Figure A (b)). For example, REST is identified for path 8, which is the neuron path, and REST is known to be required to repress neuronal gene expression in vivo [14]. ATF5/ATF7 are key regulators of nervous system development [31]. SRF, also identified by CSHMM-TF, has also been implicated in neuronal development [49]. TCF3 is a known repressor of Wnt–β-Catenin signaling and maintains neural stem cell population during neocortical development [43]. CREM is identified in path 8. Studies indicates that the lack of CREB/CREM genes leads to migration abnormalities during brain development [20]. NF1 controls neural stem cell (NSC) proliferation [13], SOX4/SOX11/SOX12 have been reported to be essential for NPC proliferation and differentiation [46].

Supporting results of large simulated liver data

See Figure D and Table D for the result cell trajectories and TF assignment. As for the results, comparing the structure for the real and simulated liver dataset, we observe that the structure and the temporal cell type assignments are overall similar, however, the larger and noisier simulated dataset does not contain some of the more detailed branching observed in the original model. This is likely the result of the increased dropout which makes it harder for the method to distinguish between similar cell types leading to them being merged in a single path. TF assignments are also pretty well conserved between the two models.

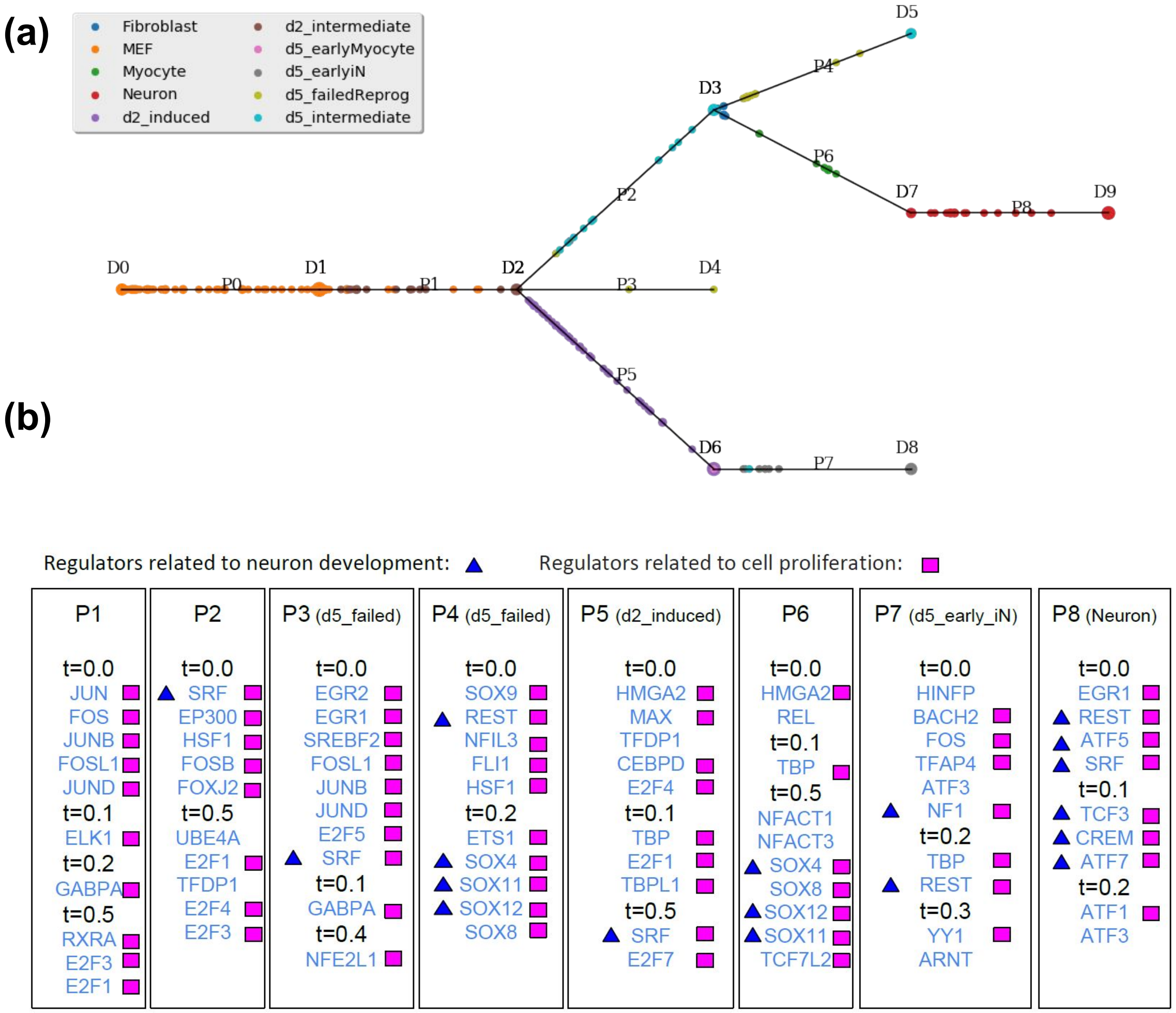

Figure A: (a) CSHMM-TF structure and continuous cell assignment for the neuron reprogramming dataset. (b) TF assignments by CSHMM-TF for the neuron reprogramming dataset. Notations are similar to the ones described in Fig. 2 in main paper

Table A: The TF assignment of SCDIFF for liver dataset. Each column shows the top 10 TFs assigned to the path based on p-values.

P1	P2	P3	P4	P5	P6	P7	P8	P9	P10	P11	P12
HMGA1	FOXO3	HMGA1	TBPL1	TBP	HMGA1	HMGA1	NKX6-2	HMGA1	TBPL1	HMGA1	HMGA1
TBPL1	NFATC3	TCF7L2	HMGA1	E2F2	E2F7	TBPL1	HMGA1	TBPL1	HMGA1	TBP	RORA
CDC5L	NFATC4	TBP	RB1	E2F5	TOPORS	TFDP1	CEBPD	PITX2	YY1	CEBPB	TBPL1
RB1	HMGA1	POU2F1	E2F1	HMGA1	E2F4	E2F4	HMGA2	ZNF350	GABPA	CEBPG	TBP
E2F7	SOX9	MTF1	NFYA	PITX2	TFDP1	NKX3-1	CEBPB	FOXO3	FOSB	MEF2A	BACH1
FOXA3	E2F7	OTX2	NFYB	SOX5	UBE4A	E2F7	FOXJ2		STAT3	HMGA2	
FOXM1	HMGA2	NKX6-2		SRY	FOXO1	E2F2	FOXD1		ATF6	NKX6-2	
SOX9		EGR1		FOXO3	E2F3	APC	CEBPG		ZNF350	SRF	
SOX11		E2F4		RB1	ATF6	RB1	SRF		STAT1	CEBPD	
SOX12		UBE4A		CD40	DSP	HMGA2	ETS1		BACH1	CDC5L	

Table B: The TF assignment for liver dataset based on the post-processing step of finding differently expressed TFs on CSHMM. Each column shows the top 10 TFs assigned to the path based on p-values.

P1	P2	P3	P4	P5	P6	P7	P8	P9	P10	P11	P12
POU5F1	POU5F1	PAX6		FOS	ZIC2	ERG	DSP	PBX1	TP53	RORA	GATA6
HNF4A	SOX11	ZIC2		HMGA2	HMGA2	DSP	ERG	NR6A1	NR6A1	MXI1	STAT3
GATA4	NR2F2	HMGA2		ZIC2	PAX6	ARID5B	FOXO1	JUND	HNF4A	RELA	EGR1
FOXA2	ZEB1	HNF1B		EGR1	OTX2	EGR1	ARID5B	HERPUD1	TCF12	CEBPD	TBPL1
PRDM1	ERG	ETV4		PAX6	HMGA1	NR2F2	STAT1	MAFF	NR1H3	CEBPB	NFE2L3
PAX6	HOXB7	OTX2		HES1	HERPUD1	FOXO1	HIF1A	CEBPD	SMAD3	TP53	
GATA6	NR6A1	HMGA1		HMGA1	MAF	TAL1	EGR1	SOX11	CREB1	STAT1	
GATA3	ELK3	PRDM1		ATF3	PITX2	STAT1	ETS1	STAT3	ETS2	ATF4	
STAT3	PBX1	GATA5		JUND	FOXM1	HMGA1	NR2F2	STAT4	IRF9	FOSB	
TP53	FLI1	GTF2I		SMAD3	NR5A2	NFE2L3	ETS2	JUN	DSP	GTF2A2	

Table C: The TF assignment to each path for myoblast dataset. Each column shows the top 10 TFs assigned to the path with assigned activation time.

P1	P2	P3	P4	P5	P6	P7	P8
IRF2 0.0	POU3F2 0.0	SRF 0.0	CDC5L 0.0	E2F1 0.0	SRF 0.0	MTF1 0.0	BPTF 0.0
TBP 0.0	ZBTB7A 0.0	BACH2 0.0	MTF1 0.0	DSP 0.0	NKX3-1 0.0	CDC5L 0.0	RFX5 0.0
POU2F1 0.0	IRF3 0.0	E2F5 0.0	TBP 0.0	TBPL1 0.0	PAX6 0.0	SRF 0.0	RFXAP 0.0
	HMGA1 0.0	E2F2 0.0	FOXO1 0.0	E2F7 0.0	MTF1 0.0	TBP 0.0	RFXANK 0.0
	NFYA 0.0	TBP 0.0	ATF6 0.0	RB1 0.0	AR 0.0	CEBPG 0.0	ZNF350 0.0
	NKX3-1 0.0	ATF1 0.0	SRF 0.0	E2F4 0.0	ZNF350 0.0	FOS 0.0	PITX2 0.0
	POU2F1 0.0	NFKB1 0.0	HMGA1 0.5	HMGA1 0.0	POU2F1 0.0	FOSL1 0.0	
		FOXO1 0.0		TFDP1 0.0	HMGA1 0.3	JUNB 0.0	
		ATF3 0.0		E2F3 0.0	TBP 0.5	JUND 0.0	
		CD40 0.1		NKX3-1 0.0	FOXO3 0.5	MEF2A 0.0	
**P9**	**P10**	**P11**	**P12**	**P13**	**P14**	**P15**	**P16**
MAX 0.0	ZBTB6 0.0	FOXJ2 0.0	FOSL1 0.0	PITX2 0.0	CEBPD 0.0	YY1 0.0	GLI2 0.0
NFYA 0.0	TBP 0.0	MITF 0.0	JUNB 0.0	SETD2 0.0	TBP 0.0	NR2F1 0.0	GLI3 0.0
NKX3-1 0.0	HMGA2 0.0	MYC 0.0	JUN 0.0	TBP 0.0	HMGA1 0.0	NR2F2 0.0	SRF 0.0
NFIC 0.0	NKX3-1 0.0	UBE4A 0.0	JUND 0.0	HIF1A 0.0	SRF 0.0	CUZD1 0.0	JUNB 0.0
VDR 0.0	POU2F1 0.0	ATF6 0.0	HMGA1 0.0	PBX1 0.0	NFATC1 0.0	RARG 0.0	JUND 0.0
		HMGA1 0.0	NR1H2 0.0		NFATC3 0.0	RARB 0.0	PBX1 0.0
		ZNF350 0.0	NR1H3 0.0		NFATC4 0.0	RARA 0.0	JUN 0.0
		NR2F2 0.0				ATF2 0.0	FOSL2 0.0
		NR1H2 0.0				ATF4 0.0	FOS 0.0
		PBX1 0.0				ATF5 0.0	POU2F1 0.0

Table D: The TF assignment to each path for simluated liver dataset ($\sim$10K cells, 20% dropout). Each column shows the top 10 TFs assigned to the path with assigned activation time. Path names are based on annotated cells assigned to that path in the figure.

P1 (DE)	P2 (HUVEC)	P3 (DE)	P4 (HE/MSC)	P5 (IH)	P6 (MSC)	P7 (MH/LB)	P8 (LB)
CDC5L 0.0	FOXJ2 0.0	NFYA 0.0	E2F3 0.0	CDC5L 0.0	TBPL1 0.0	TBPL1 0.0	HMGA1 0.0
TBPL1 0.0	HMGA1 0.0	E2F4 0.0	NFATC2 0.0	HMGA1 0.1	NKX6-2 0.0	HMGA1 0.3	SRF 0.0
HMGA1 0.0	NFATC1 0.0	E2F7 0.0	NFATC1 0.0	TOPORS 0.1	CEBPB 0.0		CEBPD 0.0
NKX3-1 0.0	NFATC2 0.0	HMGA1 0.1	NFATC4 0.0		TFDP1 0.0		TBP 0.0
ZNF219 0.0	NFATC3 0.0	TBPL1 0.3	NFATC3 0.0		DSP 0.0		CEBPG 0.0
TBP 0.1	NFATC4 0.0	DSP 0.3	E2F4 0.0		E2F4 0.0		NKX6-2 0.0
	SOX5 0.0	RB1 0.3	UBE4A 0.0		HMGA2 0.0		CDC5L 0.0
		E2F5 0.3	TBP 0.1		FOXJ2 0.0		FOXJ2 0.1
		E2F2 0.3	CEBPG 0.1		E2F7 0.1		HMGA2 0.1
		E2F1 0.5	DSP 0.5		HMGA1 0.5		GATA6 0.2

Table E: The TF assignment to each path for mouse cortical data (∼21K cells ∼10K genes). Each column shows the top 10 TFs assigned to the path with assigned activation time.

P1	P2	P3	P4
UBP1 0.0	CREB1 0.0	HLF 0.0	TBPL1 0.0
CLOCK 0.0	MYC 0.0	CLOCK 0.0	CLOCK 0.0
TFAP4 0.0	MAX 0.0	YY1 0.0	ARNT2 0.0
NFIL3 0.1	YY1 0.0	NFIL3 0.0	ELK1 0.1
ATF2 0.1	ELK1 0.0	ATF2 0.0	MEF2A 0.4
MAZ 0.2	CEBPG 0.0	SOX5 0.0	YY1 0.5
ELK1 0.2	PBX1 0.0	SOX11 0.1	
YY1 0.5	SOX11 0.0	SOX12 0.1	
	SOX12 0.0	SOX2 0.1	
	PATZ1 0.1	SOX4 0.1	

Table F: The Spearman correlation for expression of TF interactions pairs identified in Fig. 4 in main paper

Dataset	Path	TF1	TF2	Correlation
Liver	P3	sox9	nfatc1	0.52
Liver	P4	tfdp1	e2f1	0.95
Liver	P4	tfdp1	e2f3	0.74
Liver	P4	tfdp1	e2f5	0.74
Liver	P11	srf	nkx3-1	0.82
Lung	P1	yy1	atf2	0.57
Lung	P1	yy1	creb1	0.68
Lung	P1	yy1	e2f4	0.60
Lung	P2	cebpb	cebpd	0.89
Lung	P4	tfdp1	e2f4	0.67
Lung	P4	tfdp1	e2f7	0.47
Lung	P4	tfdp1	e2f2	0.44
Lung	P4	tfdp1	e2f5	0.75
Neuron	P1	jun	fos	0.18
Neuron	P1	jun	fosl1	0.31
Neuron	P2	tfdp1	e2f1	0.88
Neuron	P2	tfdp1	e2f4	0.55
Neuron	P2	tfdp1	e2f3	0.38
Neuron	P6	sox4	tcf7l2	0.43
Neuron	P6	sox11	tcf7l2	0.65
Neuron	P7	atf3	fos	0.30

Table G: The partial order list of lung/neuron/liver dataset for calculating the quantitative distance measure

**dataset**	**partial order list**
lung	(BP,AT1),(BP,AT2)
neuron	(MEF,d2_intermediate),(d2_intermediate,d5_intermediate),(d2_induced,d5_earlyiN) ,(d5_earlyiN,Neuron),(d5_earlyMyocyte,Myocyte)
liver	(iPSC,DE),(DE,HE),(IH,MH)

Table H: The quantitative distance measure reduction in % for lung/neuron/liver datasets. Larger values are better. The partial order list of each dataset are shown in Table G

**dataset**	**CSHMM-TF vs. CSHMM**	**CSHMM-TF vs. CSHMM-randomTF**
lung	9.170305677	3.711790393
neuron	1.240238861	0.780891135
liver	1.963861879	5.076180997

Table I: The comparison number of significant TF and the minimum p-value between CSHMM-TF and CSHMM-randomTF for lung/neuron/liver datasets (We define p-value ≤ 0.001 as significant here)

	**CSHMM-TF**		**CSHMM-randomTF**	
**dataset**	**# significant TF**	**min p-value**	**# significant TF**	**min p-value**
lung	44	4.74E-09	2	3.15E-04
neuron	14	3.64E-08	1	1.96E-04
liver	12	1.62E-07	6	1.44E-04

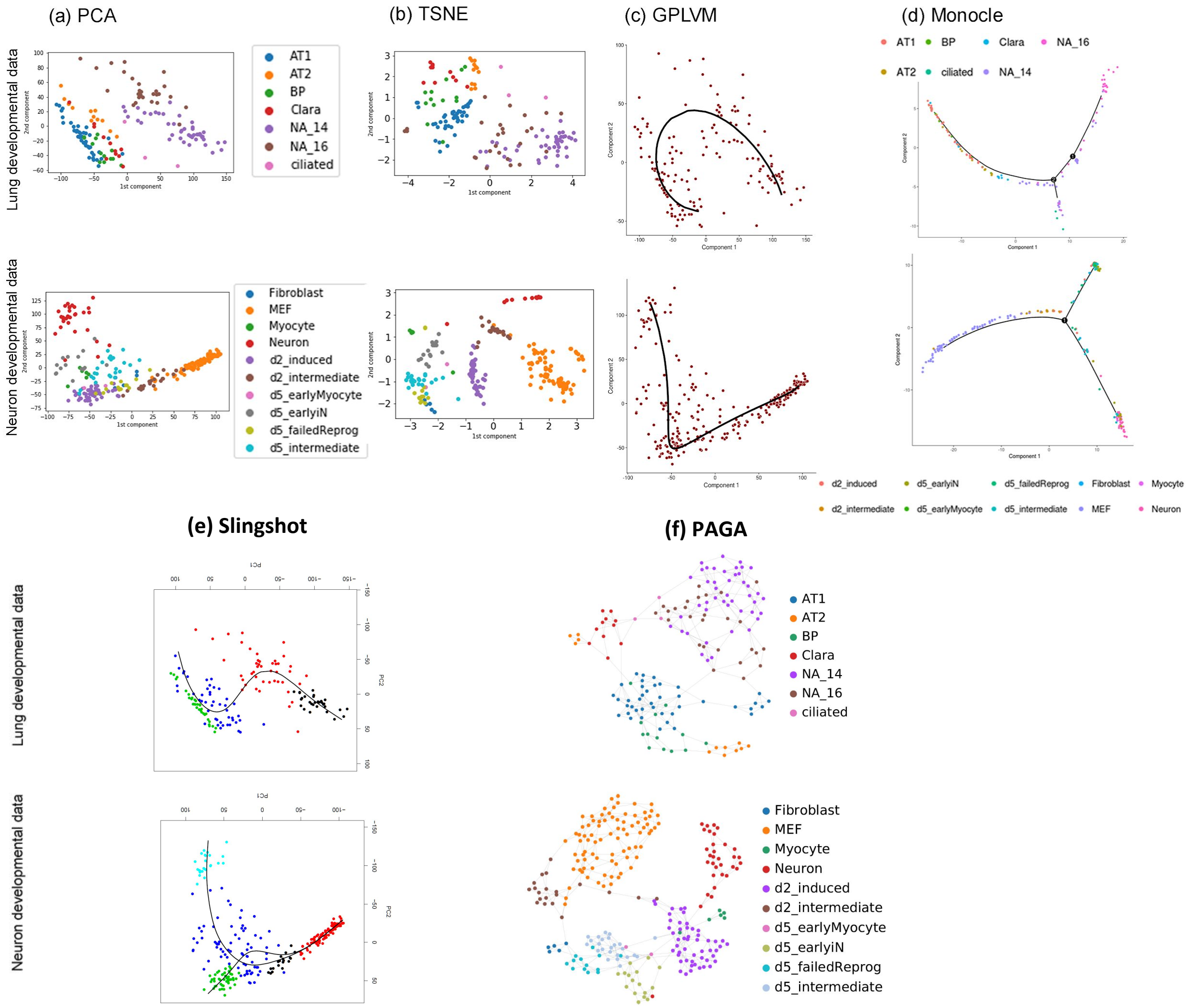

Figure B: Analysis of lung development and MEF reprogramming data by prior methods. (a) PCA (b) TSNE (c) GPLVM (d) Monocle 2 (e) Slingshot (f) PAGA. The first and the third row presents results for the lung dataset and the second and the fourth rows are for the neural developmental dataset. Colors correspond to cell fate assignments in the original papers. We run GPLVM/Slingshot/PAGA on reduced dimension by PCA. The output of GPLVM/Slingshot does not have coloring for cell types but we can see part (a) for the cell types coloring. Note: The PCA plot of Slingshot is flipped both horizontally and vertically so we also flipped it here.

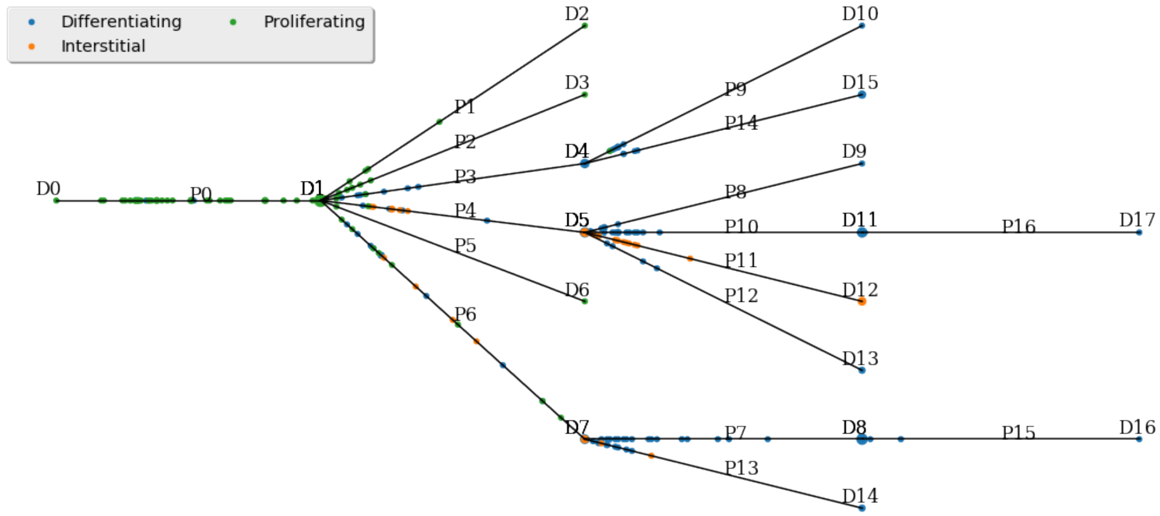

Figure C: CSHMM-TF structure and continuous cell assignment for myoblast dataset. D nodes are split nodes and p edges are paths as shown in Fig. 1 in main paper . Each circle on a path represents cells assigned to a state on that path. The bigger the circle the more cells are assigned to this state. Cells are colored based on the cell type / time point assigned to them in the original paper.

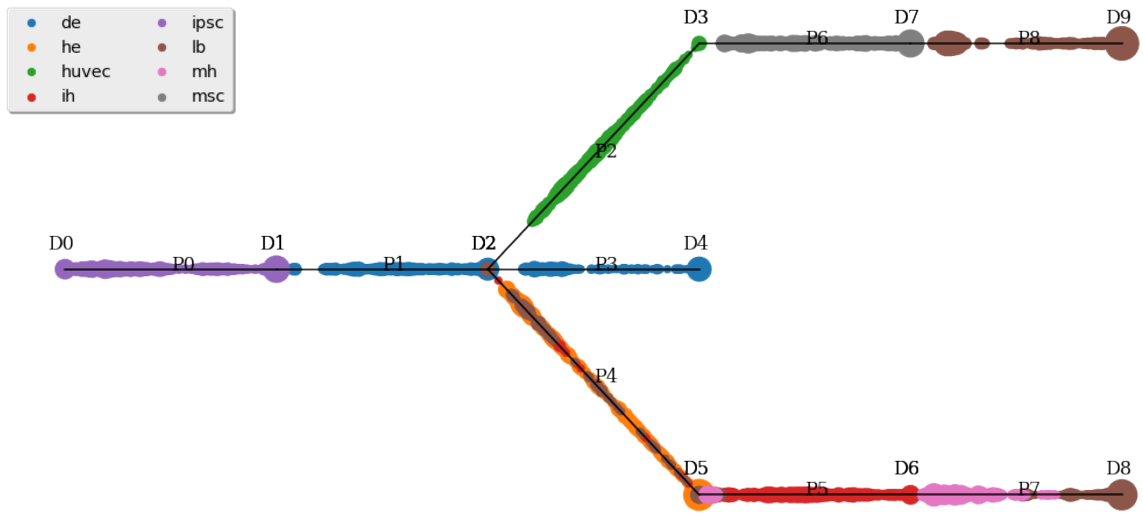

Figure D: (a) CSHMM-TF structure and continuous cell assignment for the simulated liver dataset ($\sim$ 10K cells, 20% dropout). Notations are similar to the ones described in Figure C

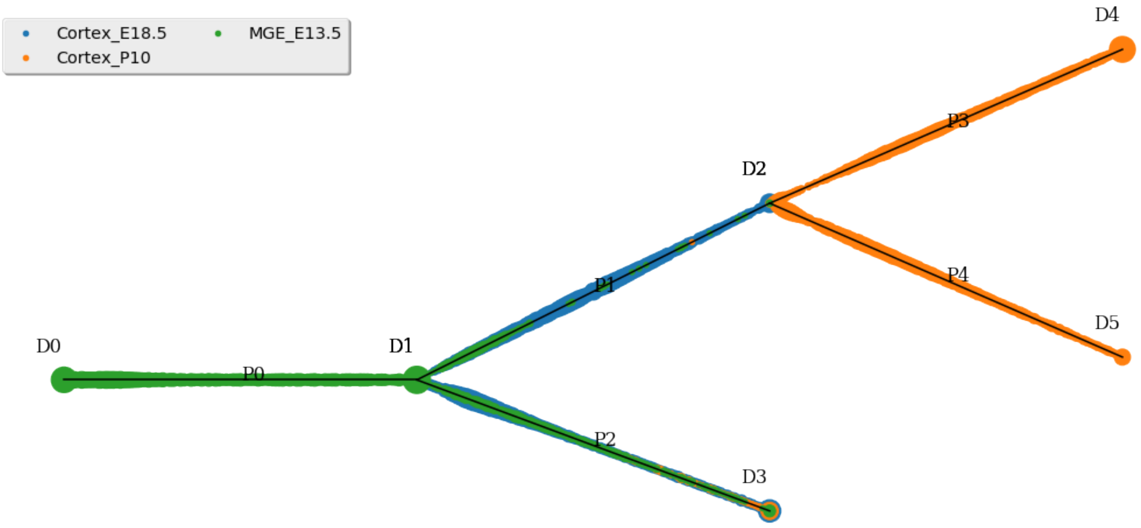

Figure E: CSHMM-TF structure and continuous cell assignment for mouse cortical dataset. Cells are labeled based on cell types and sampled time. E means embryonic days and P means postnatal days. D nodes are split nodes and p edges are paths as shown in Fig. 1 in main paper. Each circle on a path represents cells assigned to a state on that path. The bigger the circle the more cells are assigned to this state. As can be seen, the model correctly assigns cells based on their biological order (MGE-E18-P1). The model also assigns several relevant TFs to these paths as shown in Table E

.

References

[1] Ainsleigh, P. L. (2001). Theory of continuous-state hidden markov models and hidden gauss-markov models.

[2] Beuling, E., Aronson, B. E., Tran, L. M., Stapleton, K. A., ter Horst, E. N., Vissers, L. A., Verzi, M. P., and Krasinski, S. D. (2012). Gata6 is required for proliferation, migration, secretory cell maturation, and gene expression in the mature mouse colon. *Molecular and cellular biology*, pages MCB–00070.

[3] Bialkowska, A. B., Yang, V. W., and Mallipattu, S. K. (2017). Krüppel-like factors in mammalian stem cells and development. *Development*, 144(5):737–754.

[4] Birkenkamp, K. and Coffer, P. (2003). Regulation of cell survival and proliferation by the foxo (forkhead box, class o) subfamily of forkhead transcription factors.

[5] Blake, J. A., Bult, C. J., Kadin, J. A., Richardson, J. E., Eppig, J. T., and Group, M. G. D. (2010). The mouse genome database (mgd): premier model organism resource for mammalian genomics and genetics. *Nucleic acids research*, 39(suppl_1):D842–D848.

[6] Boije, M., Krajisnik, T., Jiang, Y., Kastemar, M., and Uhrbom, L. (2012). Upregulation of sox5 perturbs human glioma cell proliferation and is associated with proneural glioblastoma.

[7] Bradford, S. T., Wilhelm, D., Bandiera, R., Vidal, V., Schedl, A., and Koopman, P. (2009). A cell-autonomous role for wt1 in regulating sry in vivo. *Human molecular genetics*, 18(18):3429–3438.

[8] Bradshaw, S., Zheng, W. J., Tsoi, L. C., Gilkeson, G., and Zhang, X. K. (2008). A role for fli-1 in b cell proliferation: implications for sle pathogenesis. *Clinical Immunology*, 129(1):19–30.

[9] Bruns, H. A. and Kaplan, M. H. (2006). The role of constitutively active stat6 in leukemia and lymphoma. *Critical reviews in oncology/hematology*, 57(3):245–253.

[10] Burke, Z. D., Reed, K. R., Yeh, S.-W., Meniel, V., Sansom, O. J., Clarke, A. R., and Tosh, D. (2018). Spatiotemporal regulation of liver development by the wnt/β-catenin pathway. *Scientific reports*, 8(1):2735.

[11] Camp, J. G., Sekine, K., Gerber, T., Loeffler-Wirth, H., Binder, H., Gac, M., Kanton, S., Kageyama, J., Damm, G., Seehofer, D., et al. (2017). Multilineage communication regulates human liver bud development from pluripotency. *Nature*, 546(7659):533.

[12] Chen, D., Hu, C., Wen, G., Yang, Q., Zhang, C., and Yang, H. (2018). Downregulated sox4 expression suppresses cell proliferation, migration, and induces apoptosis in osteosarcoma in vitro and in vivo. *Calcified tissue international*, 102(1):117–127.

[13] Chen, Y.-H., Gianino, S. M., and Gutmann, D. H. (2015). Neurofibromatosis-1 regulation of neural stem cell proliferation and multilineage differentiation operates through distinct ras effector pathways. *Genes & development*.

[14] Chen, Z.-F., Paquette, A. J., and Anderson, D. J. (1998). Nrsf/rest is required in vivo for repression of multiple neuronal target genes during embryogenesis. *Nature genetics*, 20(2):136.

[15] Consortium, E. P. et al. (2007). Identification and analysis of functional elements in 1% of the human genome by the encode pilot project. *Nature*, 447(7146):799.

[16] Dai, Y., Cros, M.-P., Pontoizeau, C., Elena-Hermann, B., Bonn, G. K., and Hainaut, P. (2013). Downregulation of transcription factor e4f1 in hepatocarcinoma cells: Hbv-dependent effects on autophagy, proliferation and metabolism. *Carcinogenesis*, 35(3):635–650.

[17] D'Annibale, S., Kim, J., Magliozzi, R., Low, T. Y., Mohammed, S., Heck, A. J., and Guardavaccaro, D. (2014). Proteasome-dependent degradation of transcription factor ap4 (tfap4) controls mitotic division. *Journal of Biological Chemistry*, pages jbc–M114.

[18] Della Fazia, M. A., Servillo, G., and Sassone-Corsi, P. (1997). Cyclic amp signalling and cellular proliferation: regulation of creb and crem. *FEBS letters*, 410(1):22–24.

[19] Diamond, S. and Boyd, S. (2016). CVXPY: A Python-embedded modeling language for convex optimization. *Journal of Machine Learning Research*, 17(83):1–5.

[20] Díaz-Ruiz, C., Parlato, R., Aguado, F., Ureña, J. M., Burgaya, F., Martínez, A., Carmona, M. A., Kreiner, G., Bleckmann, S., Jose, A., et al. (2008). Regulation of neural migration by the creb/crem transcription factors and altered dab1 levels in creb/crem mutants. *Molecular and Cellular Neuroscience*, 39(4):519–528.

[21] Ding, J., Aronow, B., Kaminski, N., Kitzmiller, J., Whitsett, J., and Bar-Joseph, Z. (2018). Reconstructing differentiation networks and their regulation from time series single cell expression data. *Genome research*, pages gr–225979.

[22] Eid, M. A., Kumar, M. V., Iczkowski, K. A., Bostwick, D. G., and Tindall, D. J. (1998). Expression of early growth response genes in human prostate cancer. *Cancer research*, 58(11):2461–2468.

[23] Ernst, J., Plasterer, H. L., Simon, I., and Bar-Joseph, Z. (2010). Integrating multiple evidence sources to predict transcription factor binding in the human genome. *Genome research*.

[24] Ernst, J., Vainas, O., Harbison, C. T., Simon, I., and Bar-Joseph, Z. (2007). Reconstructing dynamic regulatory maps. *Molecular systems biology*, 3(1):74.

[25] Foletta, V. C., Segal, D. H., and Cohen, D. R. (1998). Transcriptional regulation in the immune system: all roads lead to ap-1. *Journal of Leukocyte Biology*, 63(2):139–152.

[26] Friedman, J., Hastie, T., and Tibshirani, R. (2008). Sparse inverse covariance estimation with the graphical lasso. *Biostatistics*, 9(3):432–441.

[27] Garcia-Carpizo, V., Ruiz-Llorente, S., Sarmentero, J., Graña-Castro, O., Pisano, D. G., and Barrero, M. J. (2018). Crebbp/ep300 bromodomains are critical to sustain the gata1/myc regulatory axis in proliferation. *Epigenetics & chromatin*, 11(1):30.

[28] Gordillo, M., Evans, T., and Gouon-Evans, V. (2015). Orchestrating liver development. *Development*, 142(12):2094–2108.

[29] Grant, M. and Boyd, S. (2008). Graph implementations for nonsmooth convex programs. In Blondel, V., Boyd, S., and Kimura, H., editors, *Recent Advances in Learning and Control*, Lecture Notes in Control and Information Sciences, pages 95–110. Springer-Verlag Limited. `http://stanford.edu/~boyd/graph_dcp.html`.

[30] Grant, M. and Boyd, S. (2014). CVX: Matlab software for disciplined convex programming, version 2.1. `http://cvxr.com/cvx`.

[31] Greene, L. A., Lee, H. Y., and Angelastro, J. M. (2009). The transcription factor atf5: role in neurodevelopment and neural tumors. *Journal of neurochemistry*, 108(1):11–22.

[32] Gualdrini, F., Esnault, C., Horswell, S., Stewart, A., Matthews, N., and Treisman, R. (2016). Srf co-factors control the balance between cell proliferation and contractility. *Molecular cell*, 64(6):1048–1061.

[33] Han, X., Zhang, P., Jiang, R., Xia, F., Li, M., and Guo, F.-J. (2014). Explore on the effect of atf6 on cell growth and apoptosis in cartilage development. *Histochemistry and cell biology*, 142(5):497–509.

[34] Hao, Q., Zhao, X., Zhang, Y., Dong, Z., Hu, T., and Chen, P. (2017). Targeting overexpressed activating transcription factor 1 (atf1) inhibits proliferation and migration and enhances sensitivity to paclitaxel in esophageal cancer cells. *Medical science monitor basic research*, 23:304.

[35] Hasegawa, D., Calvo, V., Avivar-Valderas, A., Lade, A., Chou, H.-I., Lee, Y. A., Farias, E. F., Aguirre-Ghiso, J. A., and Friedman, S. L. (2015). Epithelial xbp1 is required for cellular proliferation and differentiation during mammary gland development. *Molecular and cellular biology*, pages MCB–00136.

[36] Haworth, K. E., Kotecha, S., Mohun, T. J., and Latinkic, B. V. (2008). Gata4 and gata5 are essential for heart and liver development in xenopus embryos. *BMC developmental biology*, 8(1):74.

[37] Helin, K. (1998). Regulation of cell proliferation by the e2f transcription factors. *Current opinion in genetics & development*, 8(1):28–35.

[38] Indovina, P., Pentimalli, F., Casini, N., Vocca, I., and Giordano, A. (2015). Rb1 dual role in proliferation and apoptosis: cell fate control and implications for cancer therapy. *Oncotarget*, 6(20):17873.

[39] Jezierska-Drutel, A., Rosenzweig, S. A., and Neumann, C. A. (2013). Role of oxidative stress and the microenvironment in breast cancer development and progression. In *Advances in cancer research*, volume 119, pages 107–125. Elsevier.

[40] Johnson, P. F. (2005). Molecular stop signs: regulation of cell-cycle arrest by c/ebp transcription factors. *Journal of cell science*, 118(12):2545–2555.

[41] Ku, C.-C., Hasegawa, H., Lin, C.-S., Tsai, M.-H., Wuputra, K., Eckner, R., Yamaguchi, N., and Yokoyama, K. K. (2015). Control of the cell cycle and mitosis by phosphorylated activating transcription factor 2 and its homologue 7. *Journal of Nature and Science*, 1(4):e74.

[42] Kusy, S., Gerby, B., Goardon, N., Gault, N., Ferri, F., Gérard,

D., Armstrong, F., Ballerini, P., Cayuela, J.-M., Baruchel, A., et al. (2010). Nkx3. 1 is a direct tal1 target gene that mediates proliferation of tal1-expressing human t cell acute lymphoblastic leukemia. *Journal of Experimental Medicine*, pages jem–20100745.

[43] Kuwahara, A., Sakai, H., Xu, Y., Itoh, Y., Hirabayashi, Y., and Gotoh, Y. (2014). Tcf3 represses wnt–β-catenin signaling and maintains neural stem cell population during neocortical development. *PloS one*, 9(5):e94408.

[44] Li, X., Florez, S., Wang, J., Cao, H., and Amendt, B. A. (2013). Dact2 represses pitx2 transcriptional activation and cell proliferation through wnt/beta-catenin signaling during odontogenesis. *PloS one*, 8(1):e54868.

[45] Li, X., Wang, W., Xi, Y., Gao, M., Tran, M., Aziz, K. E., Qin, J., Li, W., and Chen, J. (2016). Foxr2 interacts with myc to promote its transcriptional activities and tumorigenesis. *Cell reports*, 16(2):487–497.

[46] Li, Y., Wang, J., Zheng, Y., Zhao, Y., Guo, M., Li, Y., Bao, Q., Zhang, Y., Yang, L., and Li, Q. (2012). Sox11 modulates neocortical development by regulating the proliferation and neuronal differentiation of cortical intermediate precursors. *Acta Biochim Biophys Sin*, 44(8):660–668.

[47] Lin, C. and Bar-Joseph, Z. (2019). Continuous state hmms for modeling time series single cell rna-seq data. *Bioinformatics*.

[48] Lin, K. C., Park, H. W., and Guan, K.-L. (2017). Regulation of the hippo pathway transcription factor tead. *Trends in biochemical sciences*.

[49] Lu, P. P. and Ramanan, N. (2011). Serum response factor is required for cortical axon growth but is dispensable for neurogenesis and neocortical lamination. *Journal of Neuroscience*, 31(46):16651–16664.

[50] Lu, T., Wu, B., Yu, Y., Zhu, W., Zhang, S., Zhang, Y., Guo, J., and Deng, N. (2018). Blockade of onecut2 expression in ovarian cancer inhibited tumor cell proliferation, migration, invasion and angiogenesis. *Cancer science*, 109(7):2221.

[51] Mason, J. L., Angelastro, J. M., Ignatova, T. N., Kukekov, V. G., Lin, G., Greene, L. A., and Goldman, J. E. (2005). Atf5 regulates the proliferation and differentiation of oligodendrocytes. *Molecular and Cellular Neuroscience*, 29(3):372–380.

[52] Matys, V., Kel-Margoulis, O. V., Fricke, E., Liebich, I., Land, S., Barre-Dirrie, A., Reuter, I., Chekmenev, D., Krull, M., Hornischer, K., et al. (2006). Transfac® and its module transcompel®: transcriptional gene regulation in eukaryotes. *Nucleic acids research*, 34(suppl_1):D108–D110.

[53] Mayer, C., Hafemeister, C., Bandler, R. C., Machold, R., Brito, R. B., Jaglin, X., Allaway, K., Butler, A., Fishell, G., and Satija, R. (2018). Developmental diversification of cortical inhibitory interneurons. *Nature*, 555(7697):457.

[54] Miura, Y., Morooka, M., Sax, N., Roychoudhuri, R., Itoh-Nakadai, A., Brydun, A., Funayama, R., Nakayama, K., Satomi, S., Matsumoto, M., et al. (2018). Bach2 promotes b cell receptor–induced proliferation of b lymphocytes and represses cyclin-dependent kinase inhibitors. *The Journal of Immunology*, page ji1601863.

[55] Parisi, A., Lacour, F., Giordani, L., Colnot, S., Maire, P., and Le Grand, F. (2015). Apc is required for muscle stem cell proliferation and skeletal muscle tissue repair. *J Cell Biol*, pages jcb–201501053.

[56] Patel, D. and Chaudhary, J. (2012). Increased expression of bhlh transcription factor e2a (tcf3) in prostate cancer promotes proliferation and confers resistance to doxorubicin induced apoptosis. *Biochemical and biophysical research communications*, 422(1):146–151.

[57] Poulsen, R. C., Carr, A. J., and Hulley, P. A. (2015). Cell proliferation is a key determinant of the outcome of foxo3a activation. *Biochemical and biophysical research communications*, 462(1):78–84.

[58] Rashid, S., Kotton, D. N., and Bar-Joseph, Z. (2017). Tasic: determining branching models from time series single cell data. *Bioinformatics*, page btx173.

[59] Reimold, A. M., Etkin, A., Clauss, I., Perkins, A., Friend, D. S., Zhang, J., Horton, H. F., Scott, A., Orkin, S. H., Byrne, M. C., et al. (2000). An essential role in liver development for transcription factor xbp-1. *Genes & development*, 14(2):152–157.

[60] Russell, L. and Garrett-Sinha, L. A. (2010). Transcription factor ets-1 in cytokine and chemokine gene regulation. *Cytokine*, 51(3):217–226.

[61] Satou, Y., Minami, K., Hosono, E., Okada, H., Yasuoka, Y., Shibano, T., Tanaka, T., and Taira, M. (2018). Phosphorylation states change otx2 activity for cell proliferation and patterning in the xenopus embryo. *Development*, pages dev–159640.

[62] Schulz, M. H., Devanny, W. E., Gitter, A., Zhong, S., Ernst, J., and Bar-Joseph, Z. (2012). Drem 2.0: Improved reconstruction of dynamic regulatory networks from time-series expression data. *BMC systems biology*, 6(1):104.

[63] Seal, R. L., Gordon, S. M., Lush, M. J., Wright, M. W., and Bruford, E. A. (2010). genenames. org: the hgnc resources in 2011. *Nucleic acids research*, 39(suppl_1):D514–D519.

[64] Shan, Y., Chang, T., Shi, S., Tang, M., Bao, L., Li, L., You, B., and You, Y. (2017). Foxj2 overexpression is associated with poor prognosis, progression, and metastasis in nasopharyngeal carcinoma. *OncoTargets and therapy*, 10:3733.

[65] Shu, L., Zien, K., Gutjahr, G., Oberholzer, J., Pattou, F., Kerr-Conte, J., and Maedler, K. (2012). Tcf7l2 promotes beta cell regeneration in human and mouse pancreas. *Diabetologia*, 55(12):3296–3307.

[66] Srivastava, J., Robertson, C. L., Rajasekaran, D., Gredler, R., Siddiq, A., Emdad, L., Mukhopadhyay, N. D., Ghosh, S., Hylemon, P. B., Gil, G., et al. (2014). Aeg-1 regulates retinoid x receptor and inhibits retinoid signaling. *Cancer research*, 74(16):4364–4377.

[67] Su, L., Chang, C., Han, H., Ma, H., and Xu, C. (2006). Analysis of changes about hsbp1, hsf1, hsf2 and hsp70's expression levels in rat's regenerating liver. *Fen zi xi bao sheng wu xue bao= Journal of molecular cell biology*, 39(3):258–264.

[68] Takashima, Y., Horisawa, K., Udono, M., Ohkawa, Y., and Suzuki, A. (2018). Prolonged inhibition of hepatocellular carcinoma cell proliferation by combinatorial expression of defined transcription factors. *Cancer Science*, 109(11):3543.

[69] Tan, L., Wei, X., Zheng, L., Zeng, J., Liu, H., Yang, S., and Tan, H. (2016). Amplified hmga2 promotes cell growth by regulating akt pathway in aml. *Journal of cancer research and clinical oncology*, 142(2):389–399.

[70] Tiwari, A., Swamy, S., Gopinath, K. S., and Kumar, A. (2015). Genomic amplification upregulates estrogen-related receptor alpha and its depletion inhibits oral squamous cell carcinoma tumors in vivo. *Scientific reports*, 5:17621.

[71] Trapnell, C., Cacchiarelli, D., Grimsby, J., Pokharel, P., Li, S., Morse, M., Lennon, N. J., Livak, K. J., Mikkelsen, T. S., and Rinn, J. L. (2014). The dynamics and regulators of cell fate decisions are revealed by pseudotemporal ordering of single cells. *Nature biotechnology*, 32(4):381–386.

[72] Treutlein, B., Brownfield, D. G., Wu, A. R., Neff, N. F., Mantalas, G. L., Espinoza, F. H., Desai, T. J., Krasnow, M. A., and Quake, S. R. (2014). Reconstructing lineage hierarchies of the distal lung epithelium using single cell rna-seq. *Nature*, 509(7500):371.

[73] Treutlein, B., Lee, Q. Y., Camp, J. G., Mall, M., Koh, W., Shariati, S. A. M., Sim, S., Neff, N. F., Skotheim, J. M., Wernig, M., et al. (2016). Dissecting direct reprogramming from fibroblast to neuron using single-cell rna-seq. *Nature*, 534(7607):391.

[74] Tsai, F.-Y. and Orkin, S. H. (1997). Transcription factor gata-2 is required for proliferation/survival of early hematopoietic cells and mast cell formation, but not for erythroid and myeloid terminal differentiation. *Blood*, 89(10):3636–3643.

[75] Vlieghe, D., Sandelin, A., De Bleser, P. J., Vleminckx, K., Wasserman, W. W., Van Roy, F., and Lenhard, B. (2006). A new generation of jaspar, the open-access repository for transcription factor binding site profiles. *Nucleic acids research*, 34(suppl_1):D95–D97.

[76] Wan, C., Yuan, G., Luo, D., Zhang, L., Lin, H., Liu, H., Chen, L., Yang, G., Chen, S., and Chen, Z. (2016). The dentin sialoprotein (dsp) domain regulates dental mesenchymal cell differentiation through a novel surface receptor. *Scientific reports*, 6:29666.

[77] Wang, T., Zhao, H., Gao, H., Zhu, C., Xu, Y., Bai, L., Liu, J., and Yan, F. (2018). Expression and phosphorylation of foxo1 influences cell proliferation and apoptosis in the gastrointestinal stromal tumor cell line gist-t1. *Experimental and therapeutic medicine*, 15(4):3197–3202.

[78] Wang, X., Ju, Y., Zhou, M., Liu, X., and Zhou, C. (2015). Upregulation of sox9 promotes cell proliferation, migration and invasion in lung adenocarcinoma. *Oncology letters*, 10(2):990–994.

[79] Wang, Y., Chang, H., Gao, D., Wang, L., Jiang, N., and Yu, B. (2016). Cdc5l contributes to malignant cell proliferation in human osteosarcoma via cell cycle regulation. *International Journal of Clinical & Experimental Medicine*, 9(10).

[80] Wen, Y.-A., Xiong, X., Zaytseva, Y. Y., Napier, D. L., Vallee, E., Li, A. T., Wang, C., Weiss, H. L., Evers, B. M., and Gao, T. (2018). Downregulation of srebp inhibits tumor growth and

initiation by altering cellular metabolism in colon cancer. *Cell death & disease*, 9(3):265.

[81] Wu, B., Wang, Y., Wang, C., Wang, G. G., Wu, J., and Wan, Y. Y. (2016). Bptf is essential for t cell homeostasis and function. *The Journal of Immunology*, page 1600642.

[82] Wu, S., Wang, H., Li, Y., Xie, Y., Huang, C., Zhao, H., Miyagishi, M., and Kasim, V. (2018). Transcription factor yy1 promotes cell proliferation by directly activating the pentose phosphate pathway. *Cancer Research*, pages canres–4047.

[83] Xia, L., Gong, Y., Zhang, A., Cai, S., and Zeng, Q. (2016). Loss of gata5 expression due to gene promoter methylation induces growth and colony formation of hepatocellular carcinoma cells. *Oncology letters*, 11(1):861–869.

[84] Xiang, K.-M. and Li, X.-R. (2014). Mir-133b acts as a tumor suppressor and negatively regulates tbpl1 in colorectal cancer cells. *Asian Pac J Cancer Prev*, 15(8):3767–72.

[85] Xie, C., Han, Y., Liu, Y., Han, L., and Liu, J. (2014). mirna-124 down-regulates sox8 expression and suppresses cell proliferation in non-small cell lung cancer. *International journal of clinical and experimental pathology*, 7(11):7518.

[86] Xu, W., Domingues, R. G., Fonseca-Pereira, D., Ferreira, M., Ribeiro, H., Lopez-Lastra, S., Motomura, Y., Moreira-Santos, L., Bihl, F., Braud, V., et al. (2015). Nfil3 orchestrates the emergence of common helper innate lymphoid cell precursors. *Cell reports*, 10(12):2043–2054.

[87] Yang, Z.-F., Drumea, K., Cormier, J., Wang, J., Zhu, X., and Rosmarin, A. G. (2011). Gabp transcription factor is required for myeloid differentiation, in part, through its control of gfi-1 expression. *Blood*, pages blood–2010.

[88] Zhang, D., Li, Y., Wang, R., Li, Y., Shi, P., Kan, Z., and Pang, X. (2016a). Inhibition of rest suppresses proliferation and migration in glioblastoma cells. *International journal of molecular sciences*, 17(5):664.

[89] Zhang, X., Guo, J., Wei, X., Niu, C., Jia, M., Li, Q., and Meng, D. (2018). Bach1: Function, regulation, and involvement in disease. *Oxidative medicine and cellular longevity*, 2018.

[90] Zhang, Z., Meng, G., Wang, L., Ma, Y., and Guan, Z. (2016b). The prognostic role and reduced expression of foxj2 in human hepatocellular carcinoma. *Molecular medicine reports*, 14(1):254–262.

[91] Zhong, S., Fromm, J., and Johnson, D. L. (2007). Tbp is differentially regulated by c-jun n-terminal kinase 1 (jnk1) and jnk2 through elk-1, controlling c-jun expression and cell proliferation. *Molecular and cellular biology*, 27(1):54–64.

[92] Zhong, Y., Huang, H., Chen, M., Huang, J., Wu, Q., Yan, G.-R., and Chen, D. (2017). Pou2f1 over-expression correlates with poor prognoses and promotes cell growth and epithelial-to-mesenchymal transition in hepatocellular carcinoma. *Oncotarget*, 8(27):44082.

[93] Zhou, Z., Li, Y., Jia, Q., Wang, Z., Wang, X., Hu, J., and Xiao, J. (2017). Heat shock transcription factor 1 promotes the proliferation, migration and invasion of osteosarcoma cells. *Cell proliferation*, 50(4):e12346.
